# Discovery of Novel DPP-IV Inhibitors as Potential Candidates for the Treatment of Type 2 *Diabetes Mellitus* Predicted by 3D QSAR Pharmacophore Models, Molecular Docking and *De Novo* Evolution

**DOI:** 10.3390/molecules24162870

**Published:** 2019-08-07

**Authors:** Azizullo Musoev, Sodik Numonov, Zhuhong You, Hongwei Gao

**Affiliations:** 1 Key Laboratory of Plant Resources and Chemistry in Arid Regions, Xinjiang Technical Institute of Physics and Chemistry, Chinese Academy of Sciences, Urumqi 830011, China; 2 University of Chinese Academy of Sciences, Beijing 100190, China; 3 Research Institution “Chinese-Tajik Innovation Center for Natural Products”, Dushanbe 734063, Tajikistan; 4 School of Life Science, Ludong University, Yantai 264025, China

**Keywords:** DPP-IV, T2DM, inhibitors, pharmacophore models, docking, cross-docking

## Abstract

Dipeptidyl peptidase-IV (DPP-IV) rapidly breaks down the incretin hormones glucagon-like peptide-1 (GLP-1) and glucose-dependent insulinotropic peptide (GIP). Thus, the use of DPP-IV inhibitors to retard the degradation of endogenous GLP-1 is a possible mode of therapy correcting the defect in incretin-related physiology. The aim of this study is to find a new small molecule and explore the inhibition activity to the DPP-IV enzyme using a computer aided simulation. In this study, the predicted compounds were suggested as potent anti-diabetic candidates. Chosen structures were applied following computational strategies: The generation of the three-dimensional quantitative structure-activity relationship (3D QSAR) pharmacophore models, virtual screening, molecular docking, and *de novo* Evolution. The method also validated by performing re-docking and cross-docking studies of seven protein systems for which crystal structures were available for all bound ligands. The molecular docking experiments of predicted compounds within the binding pocket of DPP-IV were conducted. By using 25 training set inhibitors, ten pharmacophore models were generated, among which hypo1 was the best pharmacophore model with the best predictive power on account of the highest cost difference (352.03), the lowest root mean squared deviation (RMSD) (2.234), and the best correlation coefficient (0.925). Hypo1 pharmacophore model was used for virtual screening. A total of 161 compounds including 120 from the databases, 25 from the training set, 16 from the test set were selected for molecular docking. Analyzing the amino acid residues of the ligand-receptor interaction, it can be concluded that Arg125, Glu205, Glu206, Tyr547, Tyr662, and Tyr666 are the main amino acid residues. The last step in this study was *de novo* Evolution that generated 11 novel compounds. The derivative dpp4_45_Evo_1 by all scores CDOCKER_ENERGY (CDOCKER, -41.79), LigScore1 (LScore1, 5.86), LigScore2 (LScore2, 7.07), PLP1 (-112.01), PLP2 (-105.77), PMF (-162.5)—have exceeded the control compound. Thus the most active compound among 11 derivative compounds is dpp4_45_Evo_1. Additionally, for derivatives dpp4_42_Evo_1, dpp4_43_Evo2, dpp4_46_Evo_4, and dpp4_47_Evo_2, significant upward shifts were recorded. The consensus score for the derivatives of dpp4_45_Evo_1 from 1 to 6, dpp4_43_Evo2 from 4 to 6, dpp4_46_Evo_4 from 1 to 6, and dpp4_47_Evo_2 from 0 to 6 were increased. Generally, predicted candidates can act as potent occurring DPP-IV inhibitors given their ability to bind directly to the active sites of DPP-IV. Our result described that the 6 re-docked and 27 cross-docked protein-ligand complexes showed RMSD values of less than 2 Å. Further investigation will result in the development of novel and potential antidiabetic drugs.

## 1. Introduction

The epidemic of type II *Diabetes mellitus* (T2DM) has been progressing rapidly, and more than 314 million people are suffering from this disease worldwide [1]. According to the estimates of the International Diabetes Federation (IDF), by the year 2040, the total number of people with diabetes will have reached 642 million [2]. T2DM is characterized by insulin resistance, and it may be combined with relatively reduced insulin secretion [3].

There are several groups of drugs for the treatment of T2DM, and they differ in the mechanism of action: Suppressing hepatic glucose output, stimulating insulin release, mitigating glucose absorption, and increasing peripheral glucose utilization [4]. These groups include sulfonylureas, biguanides, thiazolidinediones, α-glucosidase inhibitors, and dipeptidyl peptidase-IV (DPP-IV) inhibitors. Inhibitors of DPP-IV belong to the group of stimulating insulin release and is a good class of antidiabetic drugs based on their effectiveness [5,6].

DPP-IV is a serine protease that inactivates glucagon-like peptide 1 (GLP-1) and glucose-dependent insulinotropic peptide (GIP), and both of them increase insulin secretion. GLP-1 is precisely the substrate of DPP-IV, which is a predominant incretin hormone that regulates glucose activities in a glucose-dependent manner, inhibits glucagon release, decreases gastric emptying, and promotes the regeneration and differentiation of islet β-cells. DPP-IV inhibitors increase the concentration of active GLP-1 in plasma and cause the secretion of insulin in response to an increase of blood glucose level [7,8,9]. Three-Dimensional Quantitative Structure-Activity Relationship (3D QSAR) pharmacophore modeling is capable of providing information about the structural features accountable for biological activity.

We executed computational methods including 3D QSAR pharmacophore modeling, molecular docking, virtual screening, *de novo* Evolution and multiconformational docking with the aim of finding the novel, selective and potent DPP-IV inhibitor for the treatment of diabetes. The information acquired from this study can offer vital information for the upcoming development of potent Type II anti-diabetic agents based on potential DPP-IV inhibitors.

## 2. Results and Discussion

### 2.1. Generation of Pharmacophore Models

Ten pharmacophore models were generated using 25 compounds of the training set, and they have five common features: Hydrogen bond acceptor (HBA), hydrogen bond acceptor lipid (HBA_lipid), hydrogen bond donor (HBD), hydrophobic (HY) and hydrophobic aromatic (HYAr). Table S1 displays the characteristics of the 10 pharmacophore models (Hypo1 to Hypo10). The best pharmacophore model is Hypo1, which is characterized by the lowest total cost value 138.152, the highest cost difference (352.03), the lowest RMSD (2.234), and the best correlation coefficient (0.925). All the total costs were close to the fixed cost and far from the null cost. The correlation coefficient of the 10 pharmacophore models ranged from 0.925 to 0.839. ∆Cost (Null cost–Total cost) indicated the probability of representing a true correlation of data. The null cost of the ten established pharmacophore models was 490.185 bits and the fixed cost was 75.661. The difference between null and fixed costs was 414.52 bits, indicating that all 10 pharmacophore models exhibited strong predictive capacity. The configuration cost was 15.36 bits, which was in the allowed range of fewer than 17 bits.

**Figure 1:**
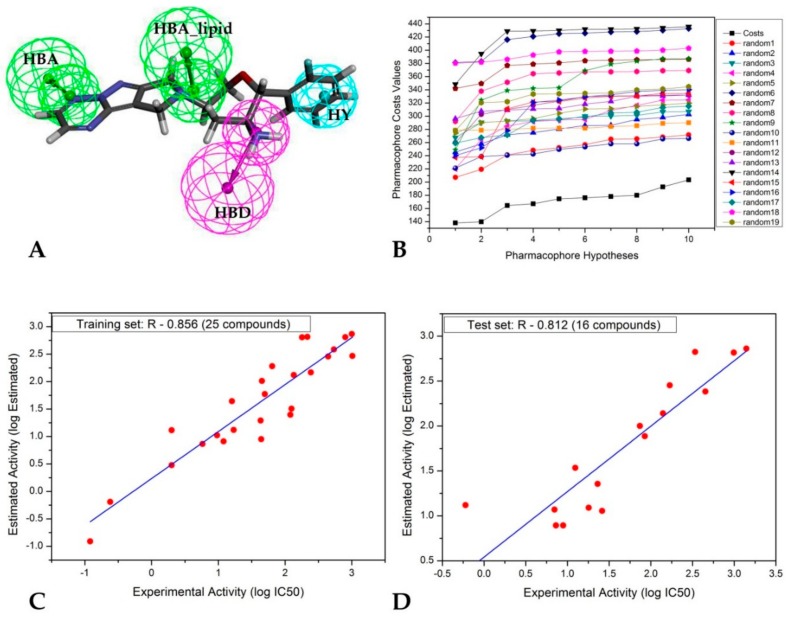
Graphical representation of pharmacophore data. (**A**) The top scoring Hypo1 is mapped to the most active compound in the training set (DPP4_1) (HBA, hydrogen bond acceptor; HBA_lipid, hydrogen bond acceptor lipid; HBD, hydrogen bond donor; HY, hydrophobic). (**B**) Fischer validation: The total cost of the initial and the 19 random spreadsheets on the 95% confidence level. (**C**) Correlation between the experimental and predicted activity (pIC_50_) by Hypo1 for the training set compounds. (**D**) Correlation between the experimental and predicted activity (pIC_50_) by Hypo1 for the test set compounds.

As presented in Figure 1A, the top scoring Hypo1 is mapped to the most active compound dpp4_1 in the training set, including four features—one hydrogen bond acceptor and one hydrogen bond acceptor lipid shown in green, one hydrophobic shown in cyan and one hydrogen bond donor shown in magenta. In compound dpp4_1, the hydrogen-bond acceptor was mapped to the 1,2,4-amino triazine and the hydrogen-bond acceptor lipid feature was mapped to the tetrahydro-2H-pyran; the hydrophobic feature was mapped to 2,5-difluorophenyl; the hydrogen bond donor feature was mapped to the nitrogen of tetrahydro-2H-pyran-3-amine.

### 2.2. Validation of Pharmacophore Models

#### 2.2.1. Training and Test Sets

The best pharmacophore model, Hypo1, was further validated by the test and training sets. The test set method is for examining whether the pharmacophore model is capable of predicting the activities of external compounds at the test set series. Twenty-five compounds were chosen as the training set to generate the pharmacophore model. The test set contained 16 compounds structurally distinct from the training set molecules. The experimental and predicted activities (IC_50_) of the training and test set compounds are listed in Table 1 and Table 2. 

Experimental (logActiv) and predicted activity (logEstimate) of the training and test set compounds are displayed in Figure 1C,D respectively.

Compounds were placed into three groups based on their activity (IC_50_): Highly active (IC_50_ < 10 nM = + + +); moderately active (10 nM ≤ IC_50_ < 100 nM = + +); less active or inactive (IC_50_ > 100 nM = +), are shown in Table 1 and Table 2. The correlation coefficient (R) of the training and test set compounds, obtained by Hypo1, were 0.856 and 0.812, respectively (Figure 1C,D). The compounds, which were used for the 3D QSAR study, were collected from different sources. It is clear for our study that the bioactivity of various DPP-IV inhibitors from different sources studied in different conditions, can influence the correlation coefficient. The correlation coefficient of our study is relatively similar to those of some other reports elsewhere in the literature [10,11].

#### 2.2.2. Fischer’s Randomization Test

The CatScramble module within Catalyst was used to verify the quality of the pharmacophore models. Fischer’s randomization test was conducted with the 95% confidence level. These 19 random sheets develop a hypothesis using the same features that were used in the original pharmacophore hypothesis (Figure 1B, the original pharmacophore models: Costs). The results indicated Hypo1 superiority over the hypothesis of random values. From this, it appeared that the ten 3D QSAR pharmacophore models were not generated by chance.

#### 2.2.3. Virtual Screening

For virtual screening, the "Druglike (5384 compounds) [12], Traditional Chinese Medicine (TCM) (8197 compounds) [13] and scPDB (5465 compounds) [14]" databases were screened and the validated specific pharmacophore model (Hypo1) was used. This yielded a total of 6365 compounds from TCM (3117), Druglike (1112) and scPDB (2136). These 6365 compounds were filtered by Lipinski’s rule and then 3031 compounds remained (TCM (842), Druglike (1111) and scPDB (1078)) with drug-like properties. The compounds which were considered as the most active agents were estimated by activity value less than 10 nM. Finally, 120 compounds remained with activity <10 nM. In total, 161 compounds (25 from the training set, 16 from the test set, 120 from the databases) were chosen for subsequent docking studies.

### 2.3. Molecular Docking Analysis

In order to understand how these ligands bind to the enzyme and hits molecules, training set molecules were taken for docking studies using CDOCKER from the software package Discovery Studio 2016 (DS2016). Table 3 shows the results of docking and scoring functions as the CDOCKER_ENERGY (-CDOCKER), LigScore1 (LScore1), LigScore2 (LScore2), -PLP1, -PLP2, -PMF and Consensus score.

**Figure 2:**
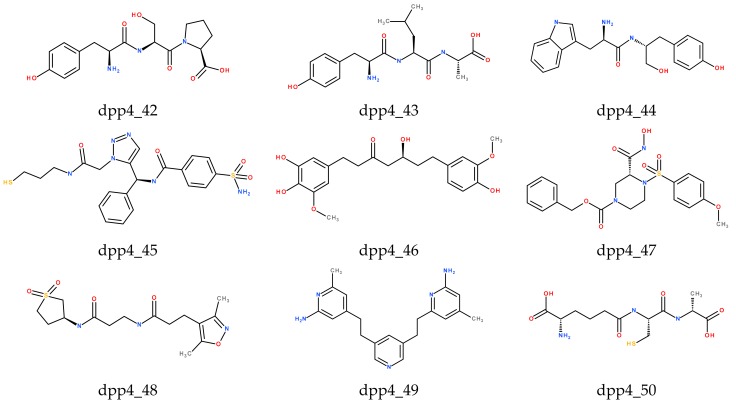

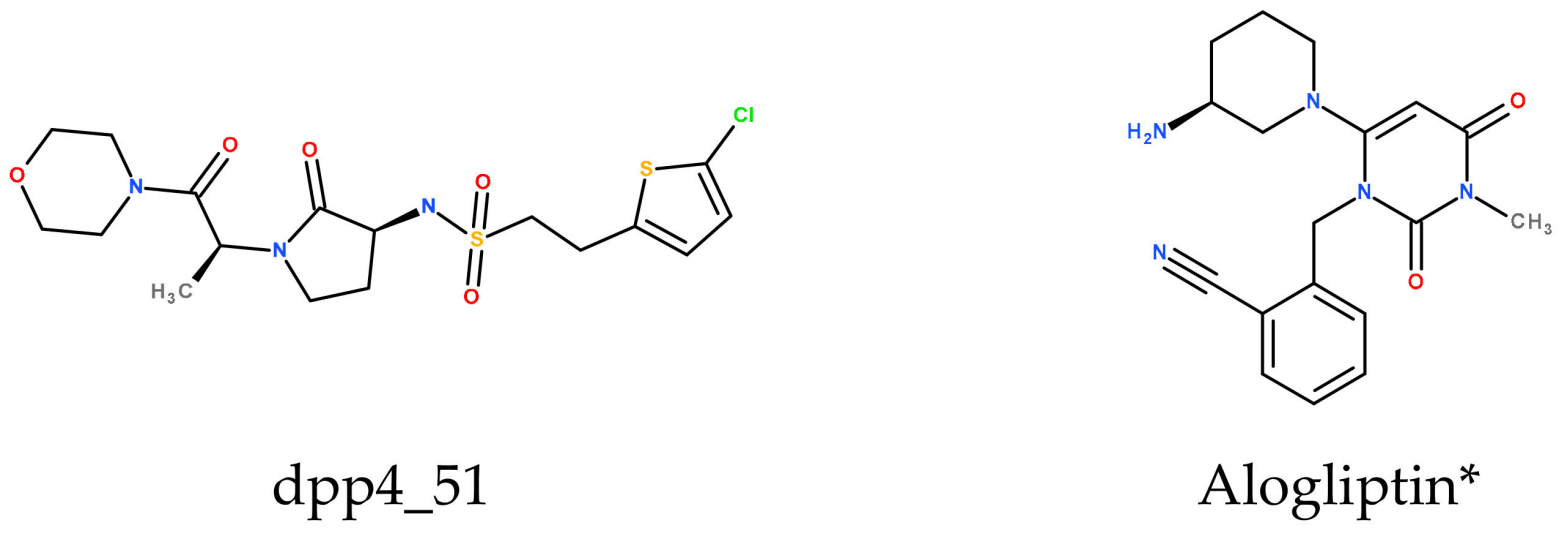
Structures of the top 11 docking compounds for DPP- IV inhibitors.

The CDOCKER score includes internal ligand strain energy and receptor–ligand interaction energy, and it is used to sort the poses of each input ligand, where a higher value indicates a more favorable binding. LScore1 and LScore2 have evaluated the polar interactions between the ligand and the receptor. The Piecewise Linear Potential (PLP) value indicates the strength of hydrogen bonds. The Potential of Mean Force (PMF) scoring function was used to examine protein–ligand binding free energies. Identification of the ligands with high scoring functions is readily apparent by the Consensus score protocol. Consensus scoring method based on different scoring functions (CDOCKER, LScore1, LScore2, PLP1, PLP2, and PMF) was applied to select the best candidate compounds from the docking study. The top 10 compounds, which have IC_50_ values of less than 10 nM with the high CDOCKER energy, are listed in Table 3. The compounds from the training set and the test set, which have relatively low CDOCKER energy and closer to the control compound (Alogliptin), but relatively low-level biological activities with very high IC50 value, have not been included in Table 3. Compounds (dpp4_42, dpp4_43, dpp4_44, dpp4_45, dpp4_47, dpp4_49, dpp4_50, dpp4_51) from the scPDB database and other two compounds from the TCM database (dpp4_46) and the Druglike database (dpp4_48) are grouped as the top 10 compounds. The CDOCKER energy of the compounds dpp4_43 and dpp4_50 are higher than all compounds, but the other scores are less than compound dpp4_42 and control compound (Alogliptin). The compound dpp4_43 has the best CDOCKER score (-83.481) among 10 candidates. Other scores—LScore1 (5.8), LScore2 (6.12), PLP1 (-87.53), PLP2 (-79.31), PMF (-136.24)—were lower than the control compound, and therefore this compound cannot be considered as a candidate. In comparison with the control compound, the CDOCKER energy and other functional scores of compound dpp4_42 are higher. Thus compound dpp4_42 resulted as the best candidate with following scores: CDOCKER (-61.722), LScore1 (6.35), LScore2 (6.47), PLP1 (-104.21), PLP2 (-103.65), PMF (-157.39). Structures of the top 11 docking compounds for DPP-IV inhibitors are presented in Figure 2.

**Figure 3:**
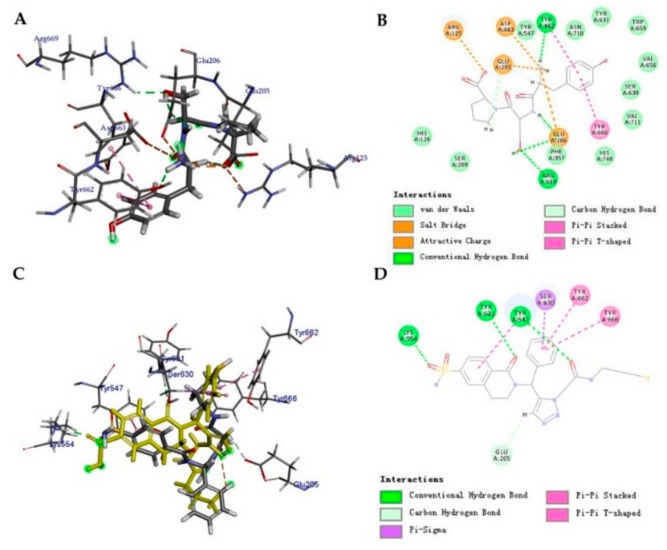
Molecular docking results. (**A**) The docking pose of dpp4_42. (**B**) The non-bonded interactions between dpp4_42 and DPP-IV. (**C**) The docking pose of dpp4_45 and derivative dpp4_45_evo_1 (colored in yellow). (**D**) The non-bonded interactions between dpp4_45_evo_1 and DPP-IV.

The docking pose of dpp4_42 and 7 key residues (Arg125, Glu205, Glu206, Tyr662, Tyr663, Tyr666, and Tyr669) are shown in Figure 3A. Figure 3B is depicted as the non-bonding interactions between the ligand dpp4_42 and protein DPP-IV. As shown in Figure 3B, non-bonded interaction between receptor and ligand occurred through hydrogen bond, hydrophobic, and electrostatic interactions. 

A summary of all docked ligands and their interacting amino acids are shown in Table S2. Based on the presented data in Table S2, we can conclude that Arg125, Glu205, Glu206, Tyr547, Tyr662, and Tyr666 are key amino acid residues of the ligand–receptor interaction.

### 2.4. Ligand de novo Evolution Analysis

**Figure 4:**
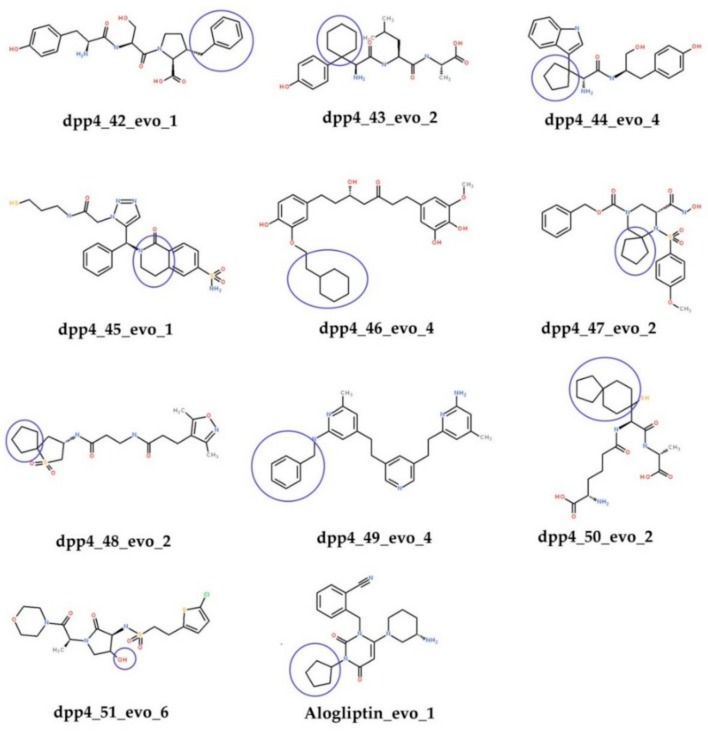
Structures of the derivatives selected after *de novo* Evolution and Molecular docking. The functional groups added in *de novo* Evolution are circled in blue.

The eleven compounds as shown in Figure 2, were run through the *de novo* Evolution protocol, and the structures of their derivatives are shown in Figure 4. The results of CDOCKER after *de novo* Evolution are tabulated in Table 4. Comparing the results with the previous docking runs (Table 3), we observed that for compound dpp4_45_Evo_1 all values CDOCKER (-41.79), LScore1 (5.86), LScore2 (7.07), PLP1 (-112.01), PLP2 (-105.77), PMF (-162.5), except CDOCKER energy were increased. All the studied values of dpp4_45_Evo_1 were higher than those of the control compound (Alogliptin) yielding dpp4_45_Evo_1 as the most active compound among 11 derivatives. Perhaps this increase in the functional scores and the consensus score is due to the addition of the piperidinone to the dpp4_45_Evo_1 molecule. All the aforementioned values also increased significantly for derivatives of dpp4_42_Evo_1, dpp4_43_Evo2, dpp4_46_Evo_4, and dpp4_47_Evo_2. The consensus score shifted from 1 to 6, from 4 to 6, from 1 to 6 and from 0 to 6 for the derivatives of dpp4_45_Evo_1, dpp4_43_Evo_2, dpp4_46_Evo_4, and dpp4_47_Evo_2, respectively. The Figure 3C,D confirms that the addition of piperidinone not only alters the binding conformation of the dpp4_45 but also enhances the interaction with the DPP-IV protein.

### 2.5. Prediction activity (IC_50_) of Compounds from Databases and their Derivatives Generated by de novo Evolution.

After molecular docking, the activities of the compounds obtained from the databases, and their derivatives, generated using *de novo* Evolution were predicted. Evaluation was performed using the best 3D QSAR pharmacophore model, Hypo1. The result of the predicted activities is presented in Table 5. The predicted activity values of the compounds dpp4_42_Evo_1, dpp4_43_Evo_2, dpp4_44_Evo_4, dpp4_45_Evo_1, dpp4_46_Evo_4, and dpp4_50_Evo_2 do not exceed 10 nM, which confirm significant activity of these compounds. This can also confirm the reliability of the results of molecular docking of the derivatives, where the above-listed compounds have good interactions with the DPP-IV protein. 

### 2.6. Calculation of RMSD of Re-Docking and Cross-Docking Tests

The performance of our re-docking and cross-docking were first evaluated by calculating the root mean square deviation (RMSD) between the predicted pose and the crystallographic ligand. Results of redocking calculation and RMSD values of seven complex protein-ligand presented in Table 6, that the six obtained RMSD values lower than 2 Å. Our method performed reasonably well and was able to produce docking poses close to X-ray confirmation. Significant results of cross-docking were observed for RMSD with values of 0.481 and 1.949 Å from 42 test which 27 of them with the values lower than 2 Å, reached 64.2 % of total test for successful predictions (Table 6).

## 3. Materials and Methods

### 3.1. Data Preparation

In this study, 41 DPP-IV inhibitors were collected from different literature sources [7,15,16,17,18,19,20,21,22]. Two-dimensional structures of the compounds were generated using the Accelrys Draw4.1 program and were saved as mol files. The structures were converted to .sdf format using the DS2016 [1]. The compounds were prepared using the “Prepare Ligand” protocol and minimized with “Full Minimization” protocol in DS2016. For the ligand-based study, 25 compounds were used as a training set (Figure S1) for generating 3D QSAR pharmacophores, and 16 compounds were used as a test set in Figure S2 for validating 3D QSAR pharmacophores [10]. The following criteria were considered in the distribution of the training set and test set: The inhibitory activity of the test set molecules lies within the activity of those of the training set, the average activity and standard deviation of both sets were close to each other, which indicated that the activity is equally distributed in the training and test sets; the sum of the inhibitory activities of the training set is relatively larger than that of the test set, suggesting that all representative points of the training set are well distributed within the entire data [23]. The range of IC_50_ values should at least span four orders of magnitude. We have selected the IC_50_ value ranges of the training set compounds from 0.12 to 1034 nM and test set from 0.6 to 1034 nM, both covering five orders of magnitude. The X-ray crystal structure of DPP-IV with the co-crystallized inhibitor alogliptin was retrieved from the RCSB Protein Data Bank (PDB: 3G0B).

### 3.2. Pharmacophore Modeling

The three-dimensional quantitative structure-activity relationship (3D-QSAR) is one of the most widely used computational methods based on chemical information in drugs development acting on targets, the structure of which is unknown [24].

The Catalyst HypoGen algorithm implemented in the DS2016 package was used for the generation of ten 3D-QSAR pharmacophore models [11]. The most important step for generating 3D-QSAR pharmacophore is selecting the chemical features. The chemical features for hypothesis generation was selected by using module feature mapping. In this study HBA, HBA_lipid, HBD, HY, and HYAr were selected with a minimum of 0 to a maximum of 5 features. The “Uncertainty” values for the training and test sets were set to 1.5, and other parameters were kept in the default value [25].

### 3.3. Pharmacophore Model Evaluation

#### 3.3.1. Cost Analysis

Cost analysis was used to select the best hypotheses from many possibilities. Two theoretical cost calculations were used to determine the quality of the pharmacophore, and they were produced by the Catalyst software package [26]. The fixed cost gives the simple model perfectly fixing all data. The null cost is the cost of a hypothesis without features, and it estimates each activity as a middling activity. The total cost should be closer to fixed cost and further away from a null cost.

#### 3.3.2. Fischer Validation

Based on this method, the correlation between the chemical structure and biological activity was verified. The method is based on randomizing activity values to the molecules in the training set. The formula used is: Value = 100 (1− (1 + x/y)), for calculating the statistical significance. Where “x” is the total number of hypotheses that contain the total costs which are lower than the most significant original hypothesis, and “y” is the number of initial HypoGen runs and random runs [26,27].

### 3.4. Virtual Screening

In order to identify novel hit compounds, the best pharmacophore model after validation was used as a 3D structural search query to screen three chemical databases—Druglike, TCM, and scPDB. Search Catalyst 3D Database module with Best/Flexible search option was applied in database screening. The hits identified through database screening were further filtered using estimated activity, Lipinski’s rule of five [28]. The Catalyst 3D Database module was used with the following parameters: Maximum hits 300, minimum inter-feature distance 0.5, which resulted in a fast search method.

### 3.5. Docking Protocol

The molecular docking was performed using program CDOCKER from the software package DS2016 [29], with the following settings: Maximum poses retained as 10.0, RMS threshold for diversity was set to 1.5, and score threshold for diversity was 20.0. The scoring functions (LigScore1, LigScore2, -PLP1, -PLP2, -PMF) were used to evaluate and rank the interactions between the ligand and the protein [30,31]. The protein crystal structure of DPP-IV (PDB ID: 3G0B) in complex with TAK-322 (Alogliptin) was taken from the Protein Data Bank. It was analyzed by the present docking study. The binding site was constructed on the basis of the binding site of alogliptin from the residues: Arg125, Glu205, Glu206, Tyr547, Tyr631, Tyr662, Tyr666 [32]. Alogliptin (activity 10 nM) is an anti-diabetic drug in the DPP-IV inhibitor class, and it was chosen as the control.

### 3.6. De Novo Evolution Protocol

The *de novo* Evolution protocol of the LUDI program is based on the initial scaffold, and it creates potential novel ligands by fitting fragments into the receptor of the binding site [10,32]. Eleven compounds were run through the protocol with the following parameters: Full Evolution mode, Evolution Scoring Function = Ludi Energy Estimate 3, Maximum RMSD = 1.0. The obtained derivatives were docked with the DPP-IV again by this protocol. The flow chart of the total experiment is shown in Figure S3.

### 3.7. Re-docking & Cross-docking Tests

To perform redocking and cross-docking calculations from Protein Data Bank (www.pdb.org) were retrieved seven x-Ray crystallographic complex protein-ligand DPP-IV with following PDB code: 2i78, 3g0b, 5j3j, 5zid, 3vjk, 5kby, 4n8d, respectively. The protein “Clean” tool in Discovery Studio was used for correcting minor problems: missing side-chain atoms which were added in an extended conformation. From each PDB file, the protein chain A was extracted for the study and water molecules were removed. The ‘Prepare Protein’ tool in Discovery Studio Accelrys, Inc (San Diego, CA92121, USA) was employed to identify problem areas in the protein structures, including missing atoms, atoms with alternate conformations, incorrectly named atoms, and incorrect bond orders. Construction of binding site was created by the following residues Arg125, Glu205, Glu206, Tyr547, Tyr631, Tyr662, Tyr666. Two structures as P_1_L_1_ and P_2_L_2_ where P_1_ and P_2_ are protein structures and L_1_ and L_2_ are corresponding ligand structures in those crystal structures, four dockings may be considered: two native dockings, P_1_L_1_ and P_2_L_2_, and two cross dockings, P_1_L_2_ and P_2_L_1_. The quality of the redocking is measured by computing the RMSD difference between computed ligand pose and the X-ray pose. In the cross dockings, a reference ligand pose is generated from the two crystal structures employed. For instance, to evaluate the cross-docking P_1_L_2_, the crystal structure P_2_L_1_ is overlaid to P_1_L_1_ by mapping the part of the protein backbone near the binding site of P_2_ to that of P_1_. The resulting overlaid pose of L_2_ is taken as the reference pose with respect to which ligand RMSD differences are computed [33].

## 4. Conclusions

In the present work, a highly correlating (r = 0.925) pharmacophore model (Hypo1) containing a hydrogen bond acceptor, a hydrogen bond acceptor lipid, a hydrogen bond donor, and a hydrophobic region feature, was selected based on various parameters such as total cost, correlation coefficient, and the cost difference. Ten 3D-QSAR pharmacophore models were obtained employing the HypoGen module; among these, Hypo1 had the best values—total cost (138.152), null cost distance (352.03), RMS (2.234) and correlation (0.925). The results of these validation tests (cost analysis, Fischer’s test) has shown that Hypo1 could accurately predict the active compounds, it has better statistical values compared to other randomly generated pharmacophore models and its correlation coefficient is not solely dependent on a single compound. Hypo1 was used for virtual screening that found 6365 drug-like compounds from the "Druglike”, “Traditional Chinese Medicine (TCM)” and “scPDB" databases. Compounds were filtered by Lipinski’s rule and selected based on which IC_50_ values less than 10 nM. The remaining 120 compounds with training (25) and test (16) sets were studied by molecular docking. Alogliptin was chosen as the control compound. Considering all scoring functions, compound dpp4_42 is the best candidate. The amino acid residues (Arg125, Glu205, Glu206, Tyr547, Tyr662, and Tyr666) involved important role in interactions between ligand–receptor. Compounds dpp4_42_Evo_1, dpp4_43_Evo2, dpp4_46_Evo_4, and dpp4_47_Evo_2 which were generated after *de novo* Evolution protocol, have significant values, and generated compound dpp4_45_Evo_1 is higher in all scores than the control compound. Therefore it can be considered the best candidate for an antidiabetic drug. To improve pose prediction and performance, we have presented RMSD values of redocking and cross-docking that confirms the validation of our investigations. The novel generated compounds can be recommended for future investigation as candidates of T2DM drugs.

## Figures and Tables

**Table 1:** The experimental DPP-IV inhibitory activities and predicted activities obtained by the HypoGen program on the basis of the pharmacophore model Hypo1 (training set).

Compound No.	Fit Value ^b^	Experimental IC_50_ nM	Predicted IC_50_ nM	Error ^a^	Experimental Scale ^c^	Predicted Scale ^c^
dpp4_1	7.91	0.12	0.12	0	+++	+++
dpp4_2	7.19	0.24	0.64	0.4	+++	+++
dpp4_3	6.52	2	2.99	0.99	+++	+++
dpp4_4	5.88	2	13	11	+++	++
dpp4_5	6.13	5.8	7.31	1.51	+++	+++
dpp4_6	5.98	9.56	10.52	0.96	+++	++
dpp4_7	6.09	12	8.15	–3.85	++	+++
dpp4_8	5.36	16	43.83	27.83	++	++
dpp4_9	5.88	17	13.16	–3.84	++	++
dpp4_10	5.71	43	19.47	–23.53	++	++
dpp4_11	6.05	44	8.94	–35.06	++	+++
dpp4_12	4.99	45	102.83	57.83	++	+
dpp4_13	5.23	49.73	59.08	9.35	++	++
dpp4_14	4.72	64.31	190.61	126.3	++	+
dpp4_15	5.60	120	24.86	–95.14	+	++
dpp4_16	5.49	125	32.05	–92.95	+	++
dpp4_17	4.88	135.45	131.69	–3.76	+	+
dpp4_18	4.19	180	639	459	+	+
dpp4_19	4.19	216.2	649.93	433.73	+	+
dpp4_20	4.83	243.67	146.8	–96.87	+	+
dpp4_21	4.54	443	285.39	–157.61	+	+
dpp4_22	4.41	540	383.37	–156.63	+	+
dpp4_23	4.19	800	644.7	–155.3	+	+
dpp4_24	4.13	1,000	736.38	–263.62	+	+
dpp4_25	4.53	1,023	292.28	–730.72	+	+

^a^ Difference between the predicted and experimental values. “+” indicates that the predicted IC_50_ is higher than the experimental IC_50_; “-“indicates that the predicted IC_50_ is lower than the experimental IC_50_. ^b^ Fit value indicates how well the features in Hypo1 overlap the chemical features in the training set compounds. ^c^ Activity scale: IC_50_ < 10 nM = +++ (highly active); 10 nM ≤ IC_50_ < 100 nM = ++ (moderately active); IC_50_ ≥ 100 nM = + (low active).

**Table 2:** The experimental dipeptidyl peptidase-IV inhibitory activities and predicted activities obtained by HypoGen program on the basis of the pharmacophore model Hypo1 (test set).

Compound No.	Fit Value ^b^	Experimental IC_50_ nM	Predicted IC_50_ nM	Error ^a^	Experimental Scale ^c^	Predicted Scale ^c^
dpp4_26	5.88	0.6	13.15	12.55	+++	++
dpp4_27	5.93	7	11.73	4.73	+++	++
dpp4_28	6.10	7.3	7.83	0.53	+++	+++
dpp4_29	6.10	8.91	7.83	–1.08	+++	+++
dpp4_30	5.46	12.45	34.24	21.79	++	++
dpp4_31	5.91	18	12.3	–5.7	++	++
dpp4_32	5.64	23	22.72	–0.28	++	++
dpp4_33	5.94	26	11.36	–14.64	++	++
dpp4_34	4.99	74	100.24	26.24	++	+
dpp4_35	5.11	84.72	76.97	–7.75	++	++
dpp4_36	4.85	140	138.08	–1.92	+	+
dpp4_37	4.55	168.63	284.03	115.4	+	+
dpp4_38	4.17	340	667.67	327.67	+	+
dpp4_39	4.62	452	241.7	–210.3	+	+
dpp4_40	4.18	990	656.11	-333.89	+	+
dpp4_41	4.14	1400	726.17	-673.83	+	+

^a^ Difference between the predicted and experimental values. “+” indicates that the predicted IC_50_ is higher than the experimental IC_50_; “-“indicates that the predicted IC_50_ is lower than the experimental IC_50_. ^b^ Fit value indicates how well the features in Hypo1 overlap the chemical features in the training set compounds. ^c^ Activity scale: IC_50_ < 10 nM = +++ (highly active); 10 nM ≤ IC_50_ < 100 nM = ++ (moderately active); IC_50_ ≥ 100 nM = + (low active).

**Table 3:** Molecular docking results of the initial compound from databases.

Rank	Name	-CDOCKER	LScore1	LScore2	-PLP1	-PLP2	-PMF	Consensus
**1**	dpp4_42	61.722	6.35	6.47	104.21	103.65	157.39	6
**2**	dpp4_43	83.481	5.8	6.12	87.53	79.31	136.24	4
**3**	dpp4_44	47.758	5.1	5.8	94.09	93.91	132.72	1
**4**	dpp4_45	46.524	4.53	5.86	103.18	86.71	119.97	1
**5**	dpp4_46	42.219	5.61	6.07	79.71	82.24	116.49	1
**6**	dpp4_47	40.196	4.14	5.61	87.6	80.08	128.71	0
**7**	dpp4_48	46.9	3.47	5.33	74.36	66.64	130.21	0
**8**	dpp4_49	43.021	4.23	5.51	75.61	69.58	109.63	0
**9**	dpp4_50	74.786	5.2	5.21	62.77	52.25	95.86	1
**10**	dpp4_51	42.476	3.53	5.74	69.71	57.31	119.7	0
**11**	Alogliptin *	40.601	3.82	6.14	96.06	92.2	142.21	4

* represent as a control compound.

**Table 4:** Molecular docking results for the derivatives generated by *de novo* Evolution protocol from top 10 DPP-IV inhibitors.

Rank	Name	-CDOCKER	LScore1	LScore2	-PLP1	-PLP2	-PMF	Consensus	LUDI3
**1**	dpp4_42_Evo_1	66.80	6.62	6.82	90.23	88.58	156.25	6	783
**2**	dpp4_43_Evo_2	76.96	6.43	6.87	98.72	90.61	152.23	6	508
**3**	dpp4_44_Evo_4	26.46	4.58	6.02	117.44	113.56	178.38	5	793
**4**	dpp4_45_Evo_1	41.79	5.86	7.07	112.01	105.77	162.5	6	592
**5**	dpp4_46_Evo_4	35.77	6.37	6.96	108.81	112.44	126.17	6	482
**6**	dpp4_47_Evo_2	15.06	5.19	6.49	104.51	91.5	147.43	6	667
**7**	dpp4_48_Evo_2	30.98	5.04	6.03	91.03	88.03	140.5	5	372
**8**	dpp4_49_Evo_4	50.86	3.69	5.88	94.79	91.65	144.29	5	850
**9**	dpp4_50_Evo_2	55.44	6.85	6.65	84.56	84.82	143.55	5	411
**10**	dpp4_51_Evo_6	43.14	5.18	6.34	77.91	80.32	116.96	5	368
**11**	Alogliptin_Evo_1	19.28	3.66	6.29	99.26	92.17	154.85	6	849

For the compound dpp4_45_Evo_1, all scores were higher than the control compound, therefore we can rate it as the best candidate for future study as an antidiabetic drug. Additionally, compounds dpp4_42_Evo_1, dpp4_43_Evo2, dpp4_46_Evo_4, and dpp4_47_Evo_2 have good indicators, and they can be studied as candidates.

**Table 5:** Results of prediction activity of compounds from databases and their derivatives.

Rank	Compounds from Databases	Fit Value	Predicted IC50 nM	Derivatives (*de novo* Evolution)	Fit Value	Predicted IC50 nM
**1**	dpp4_42	5.95	10.33	dpp4_42_Evo_1	6.08	7.66
**2**	dpp4_43	5.78	15.54	dpp4_43_Evo_2	5.51	9.91
**3**	dpp4_44	6.05	8.23	dpp4_44_Evo_4	6.49	3.00
**4**	dpp4_45	5.82	13.92	dpp4_45_Evo_1	6.00	9.25
**5**	dpp4_46	5.47	31.28	dpp4_46_Evo_4	6.20	5.79
**6**	dpp4_47	6.04	8.43	dpp4_47_Evo_2	5.88	12.32
**7**	dpp4_48	5.87	12.61	dpp4_48_Evo_2	5.80	14.72
**8**	dpp4_49	5.81	14.30	dpp4_49_Evo_4	5.57	24.77
**9**	dpp4_50	6.07	7.79	dpp4_50_Evo_2	6.04	8.36
**10**	dpp4_51	6.31	4.57	dpp4_51_Evo_6	5.95	10.48
**11**	Alogliptin	5.62	22.18	Alogliptin_Evo_1	5.56	25.26

**Table 6:** The result of RMSD values of re-docking and cross-docking calculations.

Ligand DPP-IV	2i78_l	3g0b_l	5j3j_l	5zid_l	3vjk_ l	5kby_l	4n8d_l
**2i78**	0.538	0.628	1.186	1.680	2.616	0.425	3.091
**3g0b**	0.553	0.272	1.663	1.799	3.997	0.393	2.408
**5j3j**	3.156	2.744	0.420	0.540	3.851	4.405	3.153
**5zid**	2.928	2.192	0.465	1.596	2.336	2.438	2.303
**3vjk**	0.601	2.229	0.801	0.836	0.934	4.272	2.256
**5kby**	3.137	0.642	1.557	0.646	3.851	0.698	2.378
**4n8d**	0.481	0.521	1.949	1.755	4.751	0.608	2.445

RMSD values of re-docking calculation colored in blue.

## Supplementary Materials

The following are available online at https://www.mdpi.com/1420-3049/24/16/2870/s1, Figure S1: title, Table S1: title, Video S1: title.
