# Supplementary material for: Discovery of Novel DPP-IV Inhibitors as Potential Candidates for the Treatment of Type 2 Diabetes Mellitus Predicted by 3D QSAR Pharmacophore Models, Molecular Docking and De Novo Evolution
Source: Molecules. 2019 Aug 7;24(16):2870. doi: 10.3390/molecules24162870 (PMC6720998; doi:10.3390/molecules24162870)
Supplement: Supplementary file 1 [file molecules-24-02870-s001.zip › Supplementary materials/3DQSARPharmacophoreGeneration/JobSummary.html]

1. Summary of Run Parameters:

|  |  |  |  |  |  |  |  |  |  |  |  |  |  |  |  |  |  |  |  |  |  |  |  |  |  |  |  |  |  |  |  |  |  |
| --- | --- | --- | --- | --- | --- | --- | --- | --- | --- | --- | --- | --- | --- | --- | --- | --- | --- | --- | --- | --- | --- | --- | --- | --- | --- | --- | --- | --- | --- | --- | --- | --- | --- |
| |  |  | | --- | --- | | HypoGen Parameters | | | Spacing | 150 | | Variable Weight | No | | Variable Tolerance | No | | |  |  |  | | --- | --- | --- | | Features Constraints | | | | Name | Min | Max | | HBA | 0 | 5 | | HBA\_lipid | 0 | 5 | | HBD | 0 | 5 | | HYDROPHOBIC | 0 | 5 | | HYDROPHOBAromati | 0 | 5 | | Total | 1 | 5 | |

2. Overall Results:

|  |  |  |  |  |  |  |  |  |  |  |  |  |  |  |  |  |  |  |  |  |  |  |  |  |  |  |  |  |  |  |
| --- | --- | --- | --- | --- | --- | --- | --- | --- | --- | --- | --- | --- | --- | --- | --- | --- | --- | --- | --- | --- | --- | --- | --- | --- | --- | --- | --- | --- | --- | --- |
| |  | | --- | | Pharmacophore Space: 4.221008e+04 | | Best records in pass: 5. | | Fixed Cost: 75.6612 | | Null Cost: 490.185 | | |  |  | | --- | --- | | Cost Analysis (Fixed/Null distance = 414.5 bits) | | | Index | Null Cost Distance | | 1 | 352.03 | | 2 | 350.32 | | 3 | 325.67 | | 4 | 323.08 | | 5 | 315.65 | | 6 | 313.97 | | 7 | 312.13 | | 8 | 310.16 | | 9 | 297.48 | | 10 | 286.77 | | |  |

3. Hypotheses Results:

Hypothesis 1

|  |  |  |  |  |  |  |  |  |  |  |  |  |  |  |  |  |  |  |  |  |  |  |  |  |  |  |  |  |  |  |  |  |  |  |  |  |  |  |  |  |  |  |  |  |  |  |  |  |  |  |  |  |  |  |  |  |  |  |  |  |  |  |  |  |  |  |  |  |  |  |  |  |  |  |  |  |  |  |  |  |  |  |  |  |  |  |  |  |  |  |  |  |  |  |  |  |  |  |  |  |  |  |  |  |  |  |  |  |  |  |  |  |  |  |  |  |  |  |  |  |  |  |  |  |  |  |  |  |  |  |  |  |  |  |  |  |  |  |  |  |  |  |  |  |  |  |  |  |  |  |  |  |  |  |  |  |  |  |  |  |  |  |  |  |  |  |  |  |  |  |  |  |  |  |  |  |  |  |  |  |  |  |  |  |  |  |  |  |  |  |  |  |  |  |  |  |  |  |  |  |  |  |  |
| --- | --- | --- | --- | --- | --- | --- | --- | --- | --- | --- | --- | --- | --- | --- | --- | --- | --- | --- | --- | --- | --- | --- | --- | --- | --- | --- | --- | --- | --- | --- | --- | --- | --- | --- | --- | --- | --- | --- | --- | --- | --- | --- | --- | --- | --- | --- | --- | --- | --- | --- | --- | --- | --- | --- | --- | --- | --- | --- | --- | --- | --- | --- | --- | --- | --- | --- | --- | --- | --- | --- | --- | --- | --- | --- | --- | --- | --- | --- | --- | --- | --- | --- | --- | --- | --- | --- | --- | --- | --- | --- | --- | --- | --- | --- | --- | --- | --- | --- | --- | --- | --- | --- | --- | --- | --- | --- | --- | --- | --- | --- | --- | --- | --- | --- | --- | --- | --- | --- | --- | --- | --- | --- | --- | --- | --- | --- | --- | --- | --- | --- | --- | --- | --- | --- | --- | --- | --- | --- | --- | --- | --- | --- | --- | --- | --- | --- | --- | --- | --- | --- | --- | --- | --- | --- | --- | --- | --- | --- | --- | --- | --- | --- | --- | --- | --- | --- | --- | --- | --- | --- | --- | --- | --- | --- | --- | --- | --- | --- | --- | --- | --- | --- | --- | --- | --- | --- | --- | --- | --- | --- | --- | --- | --- | --- | --- | --- | --- | --- | --- | --- | --- | --- | --- |
| |  |  |  |  |  |  |  |  |  |  |  |  |  |  |  |  |  |  |  | | --- | --- | --- | --- | --- | --- | --- | --- | --- | --- | --- | --- | --- | --- | --- | --- | --- | --- | --- | | |  |  |  |  |  |  |  |  |  |  |  |  |  |  |  |  |  |  | | --- | --- | --- | --- | --- | --- | --- | --- | --- | --- | --- | --- | --- | --- | --- | --- | --- | --- | | |  |  | | --- | --- | | Results | | | Maximum Fit | 8.38804 | | Total Cost | 138.152 | | RMS | 2.23498 | | Correlation | 0.924736 | | |  |  | | --- | --- | | Description | | | Features | Weights | | HBA  HBA\_lipid  HBD  HYDROPHOBIC | 2.09701  2.09701  2.09701  2.09701 | | | |  | | |  |  |  |  |  |  |  | | --- | --- | --- | --- | --- | --- | --- | | Name | Fit | Est | Act | Err | Status | Mapping | | dpp4\_1 | 7.9000 | 0.13000 | 0.12 | 1 | active | [25 14 13 1 ] | | dpp4\_2 | 7.1900 | 0.64000 | 0.24 | 2.7000 | active | [28 17 13 1 ] | | dpp4\_4 | 5.8800 | 13 | 2 | 6.6000 | moderately active | [30 17 \* 22 ] | | dpp4\_3 | 6.5300 | 2.9000 | 2 | 1.5000 | moderately active | [33 20 21 1 ] | | dpp4\_7 | 6.1300 | 7.3000 | 5.8 | 1.3000 | moderately active | [28 \* 21 1 ] | | dpp4\_8 | 5.9600 | 11 | 9.6 | 1.1000 | moderately active | [30 11 \* 25 ] | | dpp4\_9 | 6.0800 | 8.3000 | 12 | -1.5000 | moderately active | [\* 20 21 1 ] | | dpp4\_10 | 5.3600 | 43 | 16 | 2.7000 | moderately active | [\* 17 14 1 ] | | dpp4\_11 | 5.8900 | 13 | 17 | -1.3000 | moderately active | [\* 23 13 9 ] | | dpp4\_12 | 5.6900 | 20 | 43 | -2.1000 | moderately active | [\* 13 15 16 ] | | dpp4\_13 | 6.0500 | 8.9000 | 44 | -5 | moderately active | [\* 17 14 1 ] | | dpp4\_14 | 4.9700 | 110 | 45 | 2.4000 | moderately active | [30 17 \* 21 ] | | dpp4\_15 | 5.2300 | 59 | 50 | 1.2000 | moderately active | [26 \* 21 1 ] | | dpp4\_17 | 4.7200 | 190 | 64 | 3 | moderately active | [14 11 \* 31 ] | | dpp4\_18 | 5.6100 | 25 | 120 | -4.9000 | moderately active | [22 \* 16 43 ] | | dpp4\_19 | 5.4900 | 32 | 130 | -3.9000 | moderately active | [26 \* 15 10 ] | | dpp4\_20 | 4.9000 | 130 | 140 | -1.1000 | moderately active | [11 14 \* 25 ] | | dpp4\_21 | 4.1900 | 640 | 180 | 3.5000 | moderately active | [\* 12 \* 24 ] | | dpp4\_22 | 4.1900 | 650 | 220 | 3 | moderately active | [\* \* 21 16 ] | | dpp4\_23 | 4.8600 | 140 | 240 | -1.8000 | moderately active | [11 14 \* 25 ] | | dpp4\_24 | 4.5200 | 300 | 440 | -1.5000 | inactive | [\* 13 15 10 ] | | dpp4\_25 | 4.4300 | 370 | 540 | -1.5000 | inactive | [\* 23 13 9 ] | | dpp4\_26 | 4.1900 | 640 | 800 | -1.2000 | inactive | [\* 16 \* 20 ] | | dpp4\_27 | 4.1400 | 720 | 1000 | -1.4000 | inactive | [\* 8 \* 24 ] | | dpp4\_28 | 4.5300 | 290 | 1000 | -3.5000 | inactive | [\* 22 15 10 ] | |

Hypothesis 2

|  |  |  |  |  |  |  |  |  |  |  |  |  |  |  |  |  |  |  |  |  |  |  |  |  |  |  |  |  |  |  |  |  |  |  |  |  |  |  |  |  |  |  |  |  |  |  |  |  |  |  |  |  |  |  |  |  |  |  |  |  |  |  |  |  |  |  |  |  |  |  |  |  |  |  |  |  |  |  |  |  |  |  |  |  |  |  |  |  |  |  |  |  |  |  |  |  |  |  |  |  |  |  |  |  |  |  |  |  |  |  |  |  |  |  |  |  |  |  |  |  |  |  |  |  |  |  |  |  |  |  |  |  |  |  |  |  |  |  |  |  |  |  |  |  |  |  |  |  |  |  |  |  |  |  |  |  |  |  |  |  |  |  |  |  |  |  |  |  |  |  |  |  |  |  |  |  |  |  |  |  |  |  |  |  |  |  |  |  |  |  |  |  |  |  |  |  |  |  |  |  |  |  |  |
| --- | --- | --- | --- | --- | --- | --- | --- | --- | --- | --- | --- | --- | --- | --- | --- | --- | --- | --- | --- | --- | --- | --- | --- | --- | --- | --- | --- | --- | --- | --- | --- | --- | --- | --- | --- | --- | --- | --- | --- | --- | --- | --- | --- | --- | --- | --- | --- | --- | --- | --- | --- | --- | --- | --- | --- | --- | --- | --- | --- | --- | --- | --- | --- | --- | --- | --- | --- | --- | --- | --- | --- | --- | --- | --- | --- | --- | --- | --- | --- | --- | --- | --- | --- | --- | --- | --- | --- | --- | --- | --- | --- | --- | --- | --- | --- | --- | --- | --- | --- | --- | --- | --- | --- | --- | --- | --- | --- | --- | --- | --- | --- | --- | --- | --- | --- | --- | --- | --- | --- | --- | --- | --- | --- | --- | --- | --- | --- | --- | --- | --- | --- | --- | --- | --- | --- | --- | --- | --- | --- | --- | --- | --- | --- | --- | --- | --- | --- | --- | --- | --- | --- | --- | --- | --- | --- | --- | --- | --- | --- | --- | --- | --- | --- | --- | --- | --- | --- | --- | --- | --- | --- | --- | --- | --- | --- | --- | --- | --- | --- | --- | --- | --- | --- | --- | --- | --- | --- | --- | --- | --- | --- | --- | --- | --- | --- | --- | --- | --- | --- | --- | --- | --- | --- |
| |  |  |  |  |  |  |  |  |  |  |  |  |  |  |  |  |  |  |  | | --- | --- | --- | --- | --- | --- | --- | --- | --- | --- | --- | --- | --- | --- | --- | --- | --- | --- | --- | | |  |  |  |  |  |  |  |  |  |  |  |  |  |  |  |  |  |  | | --- | --- | --- | --- | --- | --- | --- | --- | --- | --- | --- | --- | --- | --- | --- | --- | --- | --- | | |  |  | | --- | --- | | Results | | | Maximum Fit | 8.43064 | | Total Cost | 139.866 | | RMS | 2.26525 | | Correlation | 0.922598 | | |  |  | | --- | --- | | Description | | | Features | Weights | | HBA  HBA\_lipid  HBD  HYDROPHOBIC | 2.10766  2.10766  2.10766  2.10766 | | | |  | | |  |  |  |  |  |  |  | | --- | --- | --- | --- | --- | --- | --- | | Name | Fit | Est | Act | Err | Status | Mapping | | dpp4\_1 | 7.8100 | 0.14000 | 0.12 | 1.2000 | active | [25 14 13 1 ] | | dpp4\_2 | 7.4200 | 0.35000 | 0.24 | 1.5000 | active | [25 17 13 1 ] | | dpp4\_4 | 5.8500 | 13 | 2 | 6.6000 | moderately active | [30 17 \* 22 ] | | dpp4\_3 | 6.1600 | 6.5000 | 2 | 3.2000 | moderately active | [31 20 21 1 ] | | dpp4\_7 | 6.1500 | 6.5000 | 5.8 | 1.1000 | moderately active | [27 \* 21 1 ] | | dpp4\_8 | 6.0500 | 8.2000 | 9.6 | -1.2000 | moderately active | [30 11 \* 25 ] | | dpp4\_9 | 6.1800 | 6 | 12 | -2 | moderately active | [18 20 21 1 ] | | dpp4\_10 | 5.2500 | 52 | 16 | 3.2000 | moderately active | [25 17 14 \* ] | | dpp4\_11 | 5.7300 | 17 | 17 | 1 | moderately active | [\* 23 13 9 ] | | dpp4\_12 | 5.4000 | 37 | 43 | -1.2000 | moderately active | [\* 13 15 16 ] | | dpp4\_13 | 6.0400 | 8.5000 | 44 | -5.2000 | moderately active | [\* 17 14 1 ] | | dpp4\_14 | 5.1600 | 65 | 45 | 1.4000 | moderately active | [30 17 \* 21 ] | | dpp4\_15 | 5.6200 | 22 | 50 | -2.2000 | moderately active | [26 \* 21 1 ] | | dpp4\_17 | 4.5800 | 240 | 64 | 3.8000 | moderately active | [14 11 \* 31 ] | | dpp4\_18 | 5.3700 | 40 | 120 | -3 | moderately active | [42 \* 16 15 ] | | dpp4\_19 | 5.2600 | 51 | 130 | -2.4000 | moderately active | [26 21 15 10 ] | | dpp4\_20 | 4.5900 | 240 | 140 | 1.8000 | moderately active | [11 14 \* 25 ] | | dpp4\_21 | 4.1500 | 650 | 180 | 3.6000 | moderately active | [\* 12 \* 24 ] | | dpp4\_22 | 4.2100 | 560 | 220 | 2.6000 | moderately active | [\* 21 \* 20 ] | | dpp4\_23 | 4.8000 | 150 | 240 | -1.6000 | moderately active | [11 14 \* 25 ] | | dpp4\_24 | 4.6000 | 230 | 440 | -1.9000 | inactive | [\* 13 15 10 ] | | dpp4\_25 | 4.5700 | 250 | 540 | -2.2000 | inactive | [\* 23 13 9 ] | | dpp4\_26 | 4.2000 | 590 | 800 | -1.4000 | inactive | [\* 22 \* 29 ] | | dpp4\_27 | 4.0500 | 820 | 1000 | -1.2000 | inactive | [\* 12 \* 20 ] | | dpp4\_28 | 4.6400 | 210 | 1000 | -4.8000 | inactive | [\* 13 15 10 ] | |

Hypothesis 3

|  |  |  |  |  |  |  |  |  |  |  |  |  |  |  |  |  |  |  |  |  |  |  |  |  |  |  |  |  |  |  |  |  |  |  |  |  |  |  |  |  |  |  |  |  |  |  |  |  |  |  |  |  |  |  |  |  |  |  |  |  |  |  |  |  |  |  |  |  |  |  |  |  |  |  |  |  |  |  |  |  |  |  |  |  |  |  |  |  |  |  |  |  |  |  |  |  |  |  |  |  |  |  |  |  |  |  |  |  |  |  |  |  |  |  |  |  |  |  |  |  |  |  |  |  |  |  |  |  |  |  |  |  |  |  |  |  |  |  |  |  |  |  |  |  |  |  |  |  |  |  |  |  |  |  |  |  |  |  |  |  |  |  |  |  |  |  |  |  |  |  |  |  |  |  |  |  |  |  |  |  |  |  |  |  |  |  |  |  |  |  |  |  |  |  |  |  |  |  |  |  |  |  |  |
| --- | --- | --- | --- | --- | --- | --- | --- | --- | --- | --- | --- | --- | --- | --- | --- | --- | --- | --- | --- | --- | --- | --- | --- | --- | --- | --- | --- | --- | --- | --- | --- | --- | --- | --- | --- | --- | --- | --- | --- | --- | --- | --- | --- | --- | --- | --- | --- | --- | --- | --- | --- | --- | --- | --- | --- | --- | --- | --- | --- | --- | --- | --- | --- | --- | --- | --- | --- | --- | --- | --- | --- | --- | --- | --- | --- | --- | --- | --- | --- | --- | --- | --- | --- | --- | --- | --- | --- | --- | --- | --- | --- | --- | --- | --- | --- | --- | --- | --- | --- | --- | --- | --- | --- | --- | --- | --- | --- | --- | --- | --- | --- | --- | --- | --- | --- | --- | --- | --- | --- | --- | --- | --- | --- | --- | --- | --- | --- | --- | --- | --- | --- | --- | --- | --- | --- | --- | --- | --- | --- | --- | --- | --- | --- | --- | --- | --- | --- | --- | --- | --- | --- | --- | --- | --- | --- | --- | --- | --- | --- | --- | --- | --- | --- | --- | --- | --- | --- | --- | --- | --- | --- | --- | --- | --- | --- | --- | --- | --- | --- | --- | --- | --- | --- | --- | --- | --- | --- | --- | --- | --- | --- | --- | --- | --- | --- | --- | --- | --- | --- | --- | --- | --- | --- |
| |  |  |  |  |  |  |  |  |  |  |  |  |  |  |  |  |  |  |  | | --- | --- | --- | --- | --- | --- | --- | --- | --- | --- | --- | --- | --- | --- | --- | --- | --- | --- | --- | | |  |  |  |  |  |  |  |  |  |  |  |  |  |  |  |  |  |  | | --- | --- | --- | --- | --- | --- | --- | --- | --- | --- | --- | --- | --- | --- | --- | --- | --- | --- | | |  |  | | --- | --- | | Results | | | Maximum Fit | 8.30334 | | Total Cost | 164.511 | | RMS | 2.66561 | | Correlation | 0.891029 | | |  |  | | --- | --- | | Description | | | Features | Weights | | HBA\_lipid  HBA\_lipid  HBD  HYDROPHOBIC | 2.07584  2.07584  2.07584  2.07584 | | | |  | | |  |  |  |  |  |  |  | | --- | --- | --- | --- | --- | --- | --- | | Name | Fit | Est | Act | Err | Status | Mapping | | dpp4\_1 | 7.8200 | 0.13000 | 0.12 | 1.1000 | active | [14 25 13 1 ] | | dpp4\_2 | 7.0700 | 0.75000 | 0.24 | 3.1000 | active | [17 25 13 1 ] | | dpp4\_4 | 5.9600 | 9.7000 | 2 | 4.9000 | moderately active | [17 30 \* 22 ] | | dpp4\_3 | 5.9500 | 10 | 2 | 5 | moderately active | [20 \* 21 1 ] | | dpp4\_7 | 6 | 8.8000 | 5.8 | 1.5000 | moderately active | [\* 27 21 1 ] | | dpp4\_8 | 5.9600 | 9.7000 | 9.6 | 1 | moderately active | [11 31 \* 25 ] | | dpp4\_9 | 5.9800 | 9.2000 | 12 | -1.3000 | moderately active | [20 \* 21 1 ] | | dpp4\_10 | 5.1800 | 59 | 16 | 3.7000 | moderately active | [17 \* 14 1 ] | | dpp4\_11 | 5.8700 | 12 | 17 | -1.4000 | moderately active | [23 \* 13 9 ] | | dpp4\_12 | 5.2700 | 47 | 43 | 1.1000 | moderately active | [13 \* 15 16 ] | | dpp4\_13 | 6.0500 | 7.9000 | 44 | -5.6000 | moderately active | [17 \* 14 1 ] | | dpp4\_14 | 5.0900 | 72 | 45 | 1.6000 | moderately active | [17 30 \* 21 ] | | dpp4\_15 | 4.9600 | 96 | 50 | 1.9000 | moderately active | [\* 26 21 1 ] | | dpp4\_17 | 4.5300 | 260 | 64 | 4.1000 | moderately active | [11 14 \* 31 ] | | dpp4\_18 | 5.7900 | 14 | 120 | -8.3000 | moderately active | [36 42 \* 32 ] | | dpp4\_19 | 5.5500 | 25 | 130 | -5 | moderately active | [\* 26 15 10 ] | | dpp4\_20 | 5.1200 | 68 | 140 | -2 | moderately active | [14 11 \* 25 ] | | dpp4\_21 | 4.1300 | 650 | 180 | 3.6000 | moderately active | [12 \* \* 24 ] | | dpp4\_22 | 4.1500 | 630 | 220 | 2.9000 | moderately active | [21 \* \* 20 ] | | dpp4\_23 | 5.2100 | 55 | 240 | -4.5000 | moderately active | [13 21 \* 29 ] | | dpp4\_24 | 4.5200 | 270 | 440 | -1.6000 | inactive | [13 \* 15 10 ] | | dpp4\_25 | 4.4300 | 330 | 540 | -1.6000 | inactive | [6 \* 13 19 ] | | dpp4\_26 | 4.1300 | 660 | 800 | -1.2000 | inactive | [22 \* \* 29 ] | | dpp4\_27 | 4.1000 | 710 | 1000 | -1.4000 | inactive | [8 \* \* 24 ] | | dpp4\_28 | 4.5300 | 260 | 1000 | -3.9000 | inactive | [22 \* 15 10 ] | |

Hypothesis 4

|  |  |  |  |  |  |  |  |  |  |  |  |  |  |  |  |  |  |  |  |  |  |  |  |  |  |  |  |  |  |  |  |  |  |  |  |  |  |  |  |  |  |  |  |  |  |  |  |  |  |  |  |  |  |  |  |  |  |  |  |  |  |  |  |  |  |  |  |  |  |  |  |  |  |  |  |  |  |  |  |  |  |  |  |  |  |  |  |  |  |  |  |  |  |  |  |  |  |  |  |  |  |  |  |  |  |  |  |  |  |  |  |  |  |  |  |  |  |  |  |  |  |  |  |  |  |  |  |  |  |  |  |  |  |  |  |  |  |  |  |  |  |  |  |  |  |  |  |  |  |  |  |  |  |  |  |  |  |  |  |  |  |  |  |  |  |  |  |  |  |  |  |  |  |  |  |  |  |  |  |  |  |  |  |  |  |  |  |  |  |  |  |  |  |  |  |  |  |  |  |  |  |  |  |
| --- | --- | --- | --- | --- | --- | --- | --- | --- | --- | --- | --- | --- | --- | --- | --- | --- | --- | --- | --- | --- | --- | --- | --- | --- | --- | --- | --- | --- | --- | --- | --- | --- | --- | --- | --- | --- | --- | --- | --- | --- | --- | --- | --- | --- | --- | --- | --- | --- | --- | --- | --- | --- | --- | --- | --- | --- | --- | --- | --- | --- | --- | --- | --- | --- | --- | --- | --- | --- | --- | --- | --- | --- | --- | --- | --- | --- | --- | --- | --- | --- | --- | --- | --- | --- | --- | --- | --- | --- | --- | --- | --- | --- | --- | --- | --- | --- | --- | --- | --- | --- | --- | --- | --- | --- | --- | --- | --- | --- | --- | --- | --- | --- | --- | --- | --- | --- | --- | --- | --- | --- | --- | --- | --- | --- | --- | --- | --- | --- | --- | --- | --- | --- | --- | --- | --- | --- | --- | --- | --- | --- | --- | --- | --- | --- | --- | --- | --- | --- | --- | --- | --- | --- | --- | --- | --- | --- | --- | --- | --- | --- | --- | --- | --- | --- | --- | --- | --- | --- | --- | --- | --- | --- | --- | --- | --- | --- | --- | --- | --- | --- | --- | --- | --- | --- | --- | --- | --- | --- | --- | --- | --- | --- | --- | --- | --- | --- | --- | --- | --- | --- | --- | --- | --- |
| |  |  |  |  |  |  |  |  |  |  |  |  |  |  |  |  |  |  |  | | --- | --- | --- | --- | --- | --- | --- | --- | --- | --- | --- | --- | --- | --- | --- | --- | --- | --- | --- | | |  |  |  |  |  |  |  |  |  |  |  |  |  |  |  |  |  |  | | --- | --- | --- | --- | --- | --- | --- | --- | --- | --- | --- | --- | --- | --- | --- | --- | --- | --- | | |  |  | | --- | --- | | Results | | | Maximum Fit | 8.44204 | | Total Cost | 167.104 | | RMS | 2.70372 | | Correlation | 0.887694 | | |  |  | | --- | --- | | Description | | | Features | Weights | | HBA\_lipid  HBA\_lipid  HBD  HYDROPHOBIC | 2.11051  2.11051  2.11051  2.11051 | | | |  | | |  |  |  |  |  |  |  | | --- | --- | --- | --- | --- | --- | --- | | Name | Fit | Est | Act | Err | Status | Mapping | | dpp4\_1 | 7.9200 | 0.13000 | 0.12 | 1.1000 | active | [14 25 13 1 ] | | dpp4\_2 | 7.2400 | 0.62000 | 0.24 | 2.6000 | active | [17 28 13 1 ] | | dpp4\_4 | 5.9100 | 13 | 2 | 6.5000 | moderately active | [17 30 \* 22 ] | | dpp4\_3 | 6.0200 | 10 | 2 | 5.1000 | moderately active | [20 \* 21 1 ] | | dpp4\_7 | 6.0300 | 10 | 5.8 | 1.7000 | moderately active | [\* 27 21 1 ] | | dpp4\_8 | 5.9500 | 12 | 9.6 | 1.2000 | moderately active | [11 31 \* 25 ] | | dpp4\_9 | 6.1700 | 7.2000 | 12 | -1.7000 | moderately active | [17 \* 30 1 ] | | dpp4\_10 | 5.1700 | 72 | 16 | 4.5000 | moderately active | [17 \* 14 1 ] | | dpp4\_11 | 5.8600 | 15 | 17 | -1.2000 | moderately active | [23 \* 13 9 ] | | dpp4\_12 | 5.5100 | 33 | 43 | -1.3000 | moderately active | [\* 26 15 16 ] | | dpp4\_13 | 5.9900 | 11 | 44 | -4.1000 | moderately active | [17 \* 14 1 ] | | dpp4\_14 | 5.4000 | 43 | 45 | -1.1000 | moderately active | [17 30 \* 21 ] | | dpp4\_15 | 5.6300 | 25 | 50 | -2 | moderately active | [\* 26 21 1 ] | | dpp4\_17 | 4.4300 | 400 | 64 | 6.1000 | moderately active | [11 14 \* 31 ] | | dpp4\_18 | 5.4600 | 37 | 120 | -3.2000 | moderately active | [8 16 \* 30 ] | | dpp4\_19 | 4.9800 | 110 | 130 | -1.1000 | moderately active | [13 27 \* 10 ] | | dpp4\_20 | 5.5200 | 32 | 140 | -4.3000 | moderately active | [13 21 \* 29 ] | | dpp4\_21 | 4.1900 | 680 | 180 | 3.8000 | moderately active | [12 \* \* 24 ] | | dpp4\_22 | 4.2200 | 640 | 220 | 3 | moderately active | [21 \* \* 20 ] | | dpp4\_23 | 5.2400 | 61 | 240 | -4 | moderately active | [13 21 \* 29 ] | | dpp4\_24 | 4.8000 | 170 | 440 | -2.6000 | inactive | [13 \* 15 10 ] | | dpp4\_25 | 4.3700 | 450 | 540 | -1.2000 | inactive | [6 \* 13 19 ] | | dpp4\_26 | 4.1900 | 680 | 800 | -1.2000 | inactive | [22 \* \* 29 ] | | dpp4\_27 | 4.1400 | 760 | 1000 | -1.3000 | inactive | [8 \* \* 24 ] | | dpp4\_28 | 4.9700 | 110 | 1000 | -8.9000 | inactive | [22 \* 15 10 ] | |

Hypothesis 5

|  |  |  |  |  |  |  |  |  |  |  |  |  |  |  |  |  |  |  |  |  |  |  |  |  |  |  |  |  |  |  |  |  |  |  |  |  |  |  |  |  |  |  |  |  |  |  |  |  |  |  |  |  |  |  |  |  |  |  |  |  |  |  |  |  |  |  |  |  |  |  |  |  |  |  |  |  |  |  |  |  |  |  |  |  |  |  |  |  |  |  |  |  |  |  |  |  |  |  |  |  |  |  |  |  |  |  |  |  |  |  |  |  |  |  |  |  |  |  |  |  |  |  |  |  |  |  |  |  |  |  |  |  |  |  |  |  |  |  |  |  |  |  |  |  |  |  |  |  |  |  |  |  |  |  |  |  |  |  |  |  |  |  |  |  |  |  |  |  |  |  |  |  |  |  |  |  |  |  |  |  |  |  |  |  |  |  |  |  |  |  |  |  |  |  |  |  |  |  |  |  |  |  |  |
| --- | --- | --- | --- | --- | --- | --- | --- | --- | --- | --- | --- | --- | --- | --- | --- | --- | --- | --- | --- | --- | --- | --- | --- | --- | --- | --- | --- | --- | --- | --- | --- | --- | --- | --- | --- | --- | --- | --- | --- | --- | --- | --- | --- | --- | --- | --- | --- | --- | --- | --- | --- | --- | --- | --- | --- | --- | --- | --- | --- | --- | --- | --- | --- | --- | --- | --- | --- | --- | --- | --- | --- | --- | --- | --- | --- | --- | --- | --- | --- | --- | --- | --- | --- | --- | --- | --- | --- | --- | --- | --- | --- | --- | --- | --- | --- | --- | --- | --- | --- | --- | --- | --- | --- | --- | --- | --- | --- | --- | --- | --- | --- | --- | --- | --- | --- | --- | --- | --- | --- | --- | --- | --- | --- | --- | --- | --- | --- | --- | --- | --- | --- | --- | --- | --- | --- | --- | --- | --- | --- | --- | --- | --- | --- | --- | --- | --- | --- | --- | --- | --- | --- | --- | --- | --- | --- | --- | --- | --- | --- | --- | --- | --- | --- | --- | --- | --- | --- | --- | --- | --- | --- | --- | --- | --- | --- | --- | --- | --- | --- | --- | --- | --- | --- | --- | --- | --- | --- | --- | --- | --- | --- | --- | --- | --- | --- | --- | --- | --- | --- | --- | --- | --- | --- |
| |  |  |  |  |  |  |  |  |  |  |  |  |  |  |  |  |  |  |  | | --- | --- | --- | --- | --- | --- | --- | --- | --- | --- | --- | --- | --- | --- | --- | --- | --- | --- | --- | | |  |  |  |  |  |  |  |  |  |  |  |  |  |  |  |  |  |  | | --- | --- | --- | --- | --- | --- | --- | --- | --- | --- | --- | --- | --- | --- | --- | --- | --- | --- | | |  |  | | --- | --- | | Results | | | Maximum Fit | 9.10224 | | Total Cost | 174.539 | | RMS | 2.80659 | | Correlation | 0.878395 | | |  |  | | --- | --- | | Description | | | Features | Weights | | HBA  HBA\_lipid  HBA\_lipid  HYDROPHOBIC | 2.27556  2.27556  2.27556  2.27556 | | | |  | | |  |  |  |  |  |  |  | | --- | --- | --- | --- | --- | --- | --- | | Name | Fit | Est | Act | Err | Status | Mapping | | dpp4\_1 | 8.2900 | 0.068000 | 0.12 | -1.8000 | active | [25 14 13 1 ] | | dpp4\_2 | 7.2600 | 0.74000 | 0.24 | 3.1000 | active | [28 17 13 1 ] | | dpp4\_4 | 5.9600 | 15 | 2 | 7.4000 | moderately active | [30 17 \* 22 ] | | dpp4\_3 | 6.2500 | 7.6000 | 2 | 3.8000 | moderately active | [\* 21 31 25 ] | | dpp4\_7 | 6.1200 | 10 | 5.8 | 1.7000 | moderately active | [28 \* 21 1 ] | | dpp4\_8 | 6.0900 | 11 | 9.6 | 1.1000 | moderately active | [30 11 \* 25 ] | | dpp4\_9 | 5.8800 | 18 | 12 | 1.5000 | moderately active | [\* 21 20 1 ] | | dpp4\_10 | 5.2600 | 74 | 16 | 4.6000 | moderately active | [\* 14 17 1 ] | | dpp4\_11 | 5.0300 | 120 | 17 | 7.3000 | moderately active | [\* 23 13 9 ] | | dpp4\_12 | 6.1900 | 8.7000 | 43 | -4.9000 | moderately active | [26 \* 15 16 ] | | dpp4\_13 | 6.2800 | 7 | 44 | -6.3000 | moderately active | [\* 17 14 1 ] | | dpp4\_14 | 5.5100 | 42 | 45 | -1.1000 | moderately active | [30 17 \* 21 ] | | dpp4\_15 | 5.1600 | 93 | 50 | 1.9000 | moderately active | [\* 21 8 1 ] | | dpp4\_17 | 5.0700 | 110 | 64 | 1.8000 | moderately active | [14 11 \* 31 ] | | dpp4\_18 | 5.8900 | 17 | 120 | -6.9000 | moderately active | [22 36 \* 43 ] | | dpp4\_19 | 4.9000 | 170 | 130 | 1.4000 | moderately active | [27 \* 15 10 ] | | dpp4\_20 | 4.9600 | 150 | 140 | 1.1000 | moderately active | [\* 13 11 29 ] | | dpp4\_21 | 4.5300 | 400 | 180 | 2.2000 | moderately active | [\* 12 \* 24 ] | | dpp4\_22 | 4.5400 | 380 | 220 | 1.8000 | moderately active | [\* 21 \* 20 ] | | dpp4\_23 | 5.3700 | 57 | 240 | -4.2000 | moderately active | [\* 12 14 25 ] | | dpp4\_24 | 4.7900 | 220 | 440 | -2 | inactive | [\* 13 15 10 ] | | dpp4\_25 | 4.7200 | 250 | 540 | -2.1000 | inactive | [\* 6 13 19 ] | | dpp4\_26 | 4.5300 | 400 | 800 | -2 | inactive | [\* 22 \* 29 ] | | dpp4\_27 | 4.5200 | 400 | 1000 | -2.5000 | inactive | [\* \* 8 24 ] | | dpp4\_28 | 4.7800 | 220 | 1000 | -4.6000 | inactive | [\* 22 15 10 ] | |

Hypothesis 6

|  |  |  |  |  |  |  |  |  |  |  |  |  |  |  |  |  |  |  |  |  |  |  |  |  |  |  |  |  |  |  |  |  |  |  |  |  |  |  |  |  |  |  |  |  |  |  |  |  |  |  |  |  |  |  |  |  |  |  |  |  |  |  |  |  |  |  |  |  |  |  |  |  |  |  |  |  |  |  |  |  |  |  |  |  |  |  |  |  |  |  |  |  |  |  |  |  |  |  |  |  |  |  |  |  |  |  |  |  |  |  |  |  |  |  |  |  |  |  |  |  |  |  |  |  |  |  |  |  |  |  |  |  |  |  |  |  |  |  |  |  |  |  |  |  |  |  |  |  |  |  |  |  |  |  |  |  |  |  |  |  |  |  |  |  |  |  |  |  |  |  |  |  |  |  |  |  |  |  |  |  |  |  |  |  |  |  |  |  |  |  |  |  |  |  |  |  |  |  |  |  |  |  |  |
| --- | --- | --- | --- | --- | --- | --- | --- | --- | --- | --- | --- | --- | --- | --- | --- | --- | --- | --- | --- | --- | --- | --- | --- | --- | --- | --- | --- | --- | --- | --- | --- | --- | --- | --- | --- | --- | --- | --- | --- | --- | --- | --- | --- | --- | --- | --- | --- | --- | --- | --- | --- | --- | --- | --- | --- | --- | --- | --- | --- | --- | --- | --- | --- | --- | --- | --- | --- | --- | --- | --- | --- | --- | --- | --- | --- | --- | --- | --- | --- | --- | --- | --- | --- | --- | --- | --- | --- | --- | --- | --- | --- | --- | --- | --- | --- | --- | --- | --- | --- | --- | --- | --- | --- | --- | --- | --- | --- | --- | --- | --- | --- | --- | --- | --- | --- | --- | --- | --- | --- | --- | --- | --- | --- | --- | --- | --- | --- | --- | --- | --- | --- | --- | --- | --- | --- | --- | --- | --- | --- | --- | --- | --- | --- | --- | --- | --- | --- | --- | --- | --- | --- | --- | --- | --- | --- | --- | --- | --- | --- | --- | --- | --- | --- | --- | --- | --- | --- | --- | --- | --- | --- | --- | --- | --- | --- | --- | --- | --- | --- | --- | --- | --- | --- | --- | --- | --- | --- | --- | --- | --- | --- | --- | --- | --- | --- | --- | --- | --- | --- | --- | --- | --- | --- |
| |  |  |  |  |  |  |  |  |  |  |  |  |  |  |  |  |  |  |  | | --- | --- | --- | --- | --- | --- | --- | --- | --- | --- | --- | --- | --- | --- | --- | --- | --- | --- | --- | | |  |  |  |  |  |  |  |  |  |  |  |  |  |  |  |  |  |  | | --- | --- | --- | --- | --- | --- | --- | --- | --- | --- | --- | --- | --- | --- | --- | --- | --- | --- | | |  |  | | --- | --- | | Results | | | Maximum Fit | 7.74848 | | Total Cost | 176.215 | | RMS | 2.83594 | | Correlation | 0.875645 | | |  |  | | --- | --- | | Description | | | Features | Weights | | HBA  HBA\_lipid  HBD  HYDROPHOBIC | 1.93712  1.93712  1.93712  1.93712 | | | |  | | |  |  |  |  |  |  |  | | --- | --- | --- | --- | --- | --- | --- | | Name | Fit | Est | Act | Err | Status | Mapping | | dpp4\_1 | 7.0800 | 0.27000 | 0.12 | 2.2000 | active | [25 14 13 1 ] | | dpp4\_2 | 7.1100 | 0.25000 | 0.24 | 1.1000 | active | [27 17 13 1 ] | | dpp4\_4 | 5.2600 | 18 | 2 | 8.9000 | moderately active | [\* 38 3 20 ] | | dpp4\_3 | 5.7000 | 6.5000 | 2 | 3.3000 | moderately active | [33 \* 21 1 ] | | dpp4\_7 | 5.4800 | 11 | 5.8 | 1.8000 | moderately active | [27 \* 21 1 ] | | dpp4\_8 | 5.2200 | 20 | 9.6 | 2.1000 | moderately active | [31 13 \* 25 ] | | dpp4\_9 | 5.4200 | 12 | 12 | 1 | moderately active | [\* 18 21 1 ] | | dpp4\_10 | 4.8600 | 45 | 16 | 2.8000 | moderately active | [25 17 14 \* ] | | dpp4\_11 | 4.7300 | 60 | 17 | 3.5000 | moderately active | [\* 23 13 9 ] | | dpp4\_12 | 5.0900 | 26 | 43 | -1.6000 | moderately active | [27 \* 15 12 ] | | dpp4\_13 | 5.6800 | 6.8000 | 44 | -6.5000 | moderately active | [\* 17 14 1 ] | | dpp4\_14 | 4.6800 | 68 | 45 | 1.5000 | moderately active | [28 3 \* 21 ] | | dpp4\_15 | 4.4400 | 120 | 50 | 2.3000 | moderately active | [26 \* 21 1 ] | | dpp4\_17 | 4.6500 | 73 | 64 | 1.1000 | moderately active | [14 13 \* 31 ] | | dpp4\_18 | 5.1900 | 21 | 120 | -5.7000 | moderately active | [42 36 \* 20 ] | | dpp4\_19 | 5.0500 | 29 | 130 | -4.3000 | moderately active | [27 \* 15 10 ] | | dpp4\_20 | 4.8300 | 48 | 140 | -2.9000 | moderately active | [11 14 \* 25 ] | | dpp4\_21 | 3.4600 | 1100 | 180 | 6.2000 | moderately active | [\* \* 23 19 ] | | dpp4\_22 | 3.8700 | 430 | 220 | 2 | moderately active | [\* \* 21 1 ] | | dpp4\_23 | 5.0400 | 30 | 240 | -8.2000 | moderately active | [11 14 \* 25 ] | | dpp4\_24 | 3.8700 | 430 | 440 | -1 | inactive | [\* 15 \* 23 ] | | dpp4\_25 | 4.3700 | 140 | 540 | -3.9000 | inactive | [\* 23 13 9 ] | | dpp4\_26 | 4 | 320 | 800 | -2.5000 | inactive | [21 8 16 \* ] | | dpp4\_27 | 3.4700 | 1100 | 1000 | 1.1000 | inactive | [\* \* 23 19 ] | | dpp4\_28 | 3.9000 | 410 | 1000 | -2.5000 | inactive | [\* 22 15 10 ] | |

Hypothesis 7

|  |  |  |  |  |  |  |  |  |  |  |  |  |  |  |  |  |  |  |  |  |  |  |  |  |  |  |  |  |  |  |  |  |  |  |  |  |  |  |  |  |  |  |  |  |  |  |  |  |  |  |  |  |  |  |  |  |  |  |  |  |  |  |  |  |  |  |  |  |  |  |  |  |  |  |  |  |  |  |  |  |  |  |  |  |  |  |  |  |  |  |  |  |  |  |  |  |  |  |  |  |  |  |  |  |  |  |  |  |  |  |  |  |  |  |  |  |  |  |  |  |  |  |  |  |  |  |  |  |  |  |  |  |  |  |  |  |  |  |  |  |  |  |  |  |  |  |  |  |  |  |  |  |  |  |  |  |  |  |  |  |  |  |  |  |  |  |  |  |  |  |  |  |  |  |  |  |  |  |  |  |  |  |  |  |  |  |  |  |  |  |  |  |  |  |  |  |  |  |  |  |  |  |  |
| --- | --- | --- | --- | --- | --- | --- | --- | --- | --- | --- | --- | --- | --- | --- | --- | --- | --- | --- | --- | --- | --- | --- | --- | --- | --- | --- | --- | --- | --- | --- | --- | --- | --- | --- | --- | --- | --- | --- | --- | --- | --- | --- | --- | --- | --- | --- | --- | --- | --- | --- | --- | --- | --- | --- | --- | --- | --- | --- | --- | --- | --- | --- | --- | --- | --- | --- | --- | --- | --- | --- | --- | --- | --- | --- | --- | --- | --- | --- | --- | --- | --- | --- | --- | --- | --- | --- | --- | --- | --- | --- | --- | --- | --- | --- | --- | --- | --- | --- | --- | --- | --- | --- | --- | --- | --- | --- | --- | --- | --- | --- | --- | --- | --- | --- | --- | --- | --- | --- | --- | --- | --- | --- | --- | --- | --- | --- | --- | --- | --- | --- | --- | --- | --- | --- | --- | --- | --- | --- | --- | --- | --- | --- | --- | --- | --- | --- | --- | --- | --- | --- | --- | --- | --- | --- | --- | --- | --- | --- | --- | --- | --- | --- | --- | --- | --- | --- | --- | --- | --- | --- | --- | --- | --- | --- | --- | --- | --- | --- | --- | --- | --- | --- | --- | --- | --- | --- | --- | --- | --- | --- | --- | --- | --- | --- | --- | --- | --- | --- | --- | --- | --- | --- | --- |
| |  |  |  |  |  |  |  |  |  |  |  |  |  |  |  |  |  |  |  | | --- | --- | --- | --- | --- | --- | --- | --- | --- | --- | --- | --- | --- | --- | --- | --- | --- | --- | --- | | |  |  |  |  |  |  |  |  |  |  |  |  |  |  |  |  |  |  | | --- | --- | --- | --- | --- | --- | --- | --- | --- | --- | --- | --- | --- | --- | --- | --- | --- | --- | | |  |  | | --- | --- | | Results | | | Maximum Fit | 7.34102 | | Total Cost | 178.056 | | RMS | 2.86002 | | Correlation | 0.873374 | | |  |  | | --- | --- | | Description | | | Features | Weights | | HBA  HBA\_lipid  HBD  HYDROPHOBIC | 1.83525  1.83525  1.83525  1.83525 | | | |  | | |  |  |  |  |  |  |  | | --- | --- | --- | --- | --- | --- | --- | | Name | Fit | Est | Act | Err | Status | Mapping | | dpp4\_1 | 7.0800 | 0.12000 | 0.12 | -1 | active | [25 14 13 1 ] | | dpp4\_2 | 6.7900 | 0.23000 | 0.24 | -1 | active | [28 17 13 1 ] | | dpp4\_4 | 4.8300 | 21 | 2 | 10 | moderately active | [30 17 \* 22 ] | | dpp4\_3 | 4.7400 | 26 | 2 | 13 | moderately active | [31 20 \* 1 ] | | dpp4\_7 | 5.2400 | 8.1000 | 5.8 | 1.4000 | moderately active | [28 \* 21 1 ] | | dpp4\_8 | 5.1300 | 10 | 9.6 | 1.1000 | moderately active | [30 11 \* 25 ] | | dpp4\_9 | 4.8000 | 22 | 12 | 1.9000 | moderately active | [18 20 \* 1 ] | | dpp4\_10 | 4.4800 | 47 | 16 | 2.9000 | moderately active | [25 17 14 \* ] | | dpp4\_11 | 4.1100 | 110 | 17 | 6.4000 | moderately active | [\* 23 13 9 ] | | dpp4\_12 | 4.8300 | 21 | 43 | -2 | moderately active | [26 \* 15 16 ] | | dpp4\_13 | 5.3600 | 6.2000 | 44 | -7.1000 | moderately active | [\* 17 14 1 ] | | dpp4\_14 | 4.6300 | 33 | 45 | -1.4000 | moderately active | [30 17 \* 21 ] | | dpp4\_15 | 4.5900 | 36 | 50 | -1.4000 | moderately active | [26 \* 21 1 ] | | dpp4\_17 | 3.6300 | 330 | 64 | 5.2000 | moderately active | [\* 14 \* 25 ] | | dpp4\_18 | 4.6000 | 35 | 120 | -3.4000 | moderately active | [\* 8 16 20 ] | | dpp4\_19 | 4.5500 | 40 | 130 | -3.1000 | moderately active | [26 21 15 10 ] | | dpp4\_20 | 4.3500 | 63 | 140 | -2.2000 | moderately active | [11 14 \* 25 ] | | dpp4\_21 | 3.5800 | 370 | 180 | 2.1000 | moderately active | [\* 12 \* 24 ] | | dpp4\_22 | 3.6700 | 300 | 220 | 1.4000 | moderately active | [\* \* 21 1 ] | | dpp4\_23 | 4.3800 | 59 | 240 | -4.2000 | moderately active | [11 14 \* 25 ] | | dpp4\_24 | 3.6700 | 300 | 440 | -1.5000 | inactive | [\* 15 \* 22 ] | | dpp4\_25 | 3.6700 | 300 | 540 | -1.8000 | inactive | [\* 13 \* 19 ] | | dpp4\_26 | 3.6700 | 300 | 800 | -2.6000 | inactive | [\* 21 \* 14 ] | | dpp4\_27 | 3.3900 | 580 | 1000 | -1.7000 | inactive | [\* 8 \* 24 ] | | dpp4\_28 | 3.6500 | 310 | 1000 | -3.3000 | inactive | [\* 15 \* 21 ] | |

Hypothesis 8

|  |  |  |  |  |  |  |  |  |  |  |  |  |  |  |  |  |  |  |  |  |  |  |  |  |  |  |  |  |  |  |  |  |  |  |  |  |  |  |  |  |  |  |  |  |  |  |  |  |  |  |  |  |  |  |  |  |  |  |  |  |  |  |  |  |  |  |  |  |  |  |  |  |  |  |  |  |  |  |  |  |  |  |  |  |  |  |  |  |  |  |  |  |  |  |  |  |  |  |  |  |  |  |  |  |  |  |  |  |  |  |  |  |  |  |  |  |  |  |  |  |  |  |  |  |  |  |  |  |  |  |  |  |  |  |  |  |  |  |  |  |  |  |  |  |  |  |  |  |  |  |  |  |  |  |  |  |  |  |  |  |  |  |  |  |  |  |  |  |  |  |  |  |  |  |  |  |  |  |  |  |  |  |  |  |  |  |  |  |  |  |  |  |  |  |  |  |  |  |  |  |  |  |  |
| --- | --- | --- | --- | --- | --- | --- | --- | --- | --- | --- | --- | --- | --- | --- | --- | --- | --- | --- | --- | --- | --- | --- | --- | --- | --- | --- | --- | --- | --- | --- | --- | --- | --- | --- | --- | --- | --- | --- | --- | --- | --- | --- | --- | --- | --- | --- | --- | --- | --- | --- | --- | --- | --- | --- | --- | --- | --- | --- | --- | --- | --- | --- | --- | --- | --- | --- | --- | --- | --- | --- | --- | --- | --- | --- | --- | --- | --- | --- | --- | --- | --- | --- | --- | --- | --- | --- | --- | --- | --- | --- | --- | --- | --- | --- | --- | --- | --- | --- | --- | --- | --- | --- | --- | --- | --- | --- | --- | --- | --- | --- | --- | --- | --- | --- | --- | --- | --- | --- | --- | --- | --- | --- | --- | --- | --- | --- | --- | --- | --- | --- | --- | --- | --- | --- | --- | --- | --- | --- | --- | --- | --- | --- | --- | --- | --- | --- | --- | --- | --- | --- | --- | --- | --- | --- | --- | --- | --- | --- | --- | --- | --- | --- | --- | --- | --- | --- | --- | --- | --- | --- | --- | --- | --- | --- | --- | --- | --- | --- | --- | --- | --- | --- | --- | --- | --- | --- | --- | --- | --- | --- | --- | --- | --- | --- | --- | --- | --- | --- | --- | --- | --- | --- | --- |
| |  |  |  |  |  |  |  |  |  |  |  |  |  |  |  |  |  |  |  | | --- | --- | --- | --- | --- | --- | --- | --- | --- | --- | --- | --- | --- | --- | --- | --- | --- | --- | --- | | |  |  |  |  |  |  |  |  |  |  |  |  |  |  |  |  |  |  | | --- | --- | --- | --- | --- | --- | --- | --- | --- | --- | --- | --- | --- | --- | --- | --- | --- | --- | | |  |  | | --- | --- | | Results | | | Maximum Fit | 8.0925 | | Total Cost | 180.027 | | RMS | 2.88947 | | Correlation | 0.870556 | | |  |  | | --- | --- | | Description | | | Features | Weights | | HBA  HBA\_lipid  HBD  HYDROPHOBIC | 2.02312  2.02312  2.02312  2.02312 | | | |  | | |  |  |  |  |  |  |  | | --- | --- | --- | --- | --- | --- | --- | | Name | Fit | Est | Act | Err | Status | Mapping | | dpp4\_1 | 7.0800 | 0.35000 | 0.12 | 2.9000 | active | [25 14 13 1 ] | | dpp4\_2 | 6.8900 | 0.55000 | 0.24 | 2.3000 | active | [25 17 13 1 ] | | dpp4\_4 | 5.0600 | 37 | 2 | 19 | moderately active | [\* 23 3 20 ] | | dpp4\_3 | 6.3800 | 1.8000 | 2 | -1.1000 | moderately active | [31 20 21 1 ] | | dpp4\_7 | 5.5100 | 13 | 5.8 | 2.3000 | moderately active | [27 \* 21 1 ] | | dpp4\_8 | 5.4500 | 15 | 9.6 | 1.6000 | moderately active | [31 13 \* 25 ] | | dpp4\_9 | 5.5400 | 12 | 12 | 1 | moderately active | [\* 30 21 1 ] | | dpp4\_10 | 4.4100 | 160 | 16 | 10 | moderately active | [25 \* 14 1 ] | | dpp4\_11 | 5.2300 | 25 | 17 | 1.5000 | moderately active | [\* 23 13 8 ] | | dpp4\_12 | 5.6900 | 8.6000 | 43 | -5 | moderately active | [27 \* 15 12 ] | | dpp4\_13 | 5.9200 | 5.1000 | 44 | -8.7000 | moderately active | [\* 17 14 1 ] | | dpp4\_14 | 5 | 43 | 45 | -1.1000 | moderately active | [28 3 \* 21 ] | | dpp4\_15 | 4.8700 | 57 | 50 | 1.1000 | moderately active | [26 \* 21 1 ] | | dpp4\_17 | 4.8700 | 58 | 64 | -1.1000 | moderately active | [\* 13 21 25 ] | | dpp4\_18 | 5.1800 | 28 | 120 | -4.3000 | moderately active | [22 26 \* 39 ] | | dpp4\_19 | 4.8300 | 62 | 130 | -2 | moderately active | [26 15 \* 10 ] | | dpp4\_20 | 4.8200 | 65 | 140 | -2.1000 | moderately active | [11 13 21 25 ] | | dpp4\_21 | 3.7000 | 850 | 180 | 4.7000 | moderately active | [\* \* 23 19 ] | | dpp4\_22 | 4.0400 | 390 | 220 | 1.8000 | moderately active | [\* 21 \* 25 ] | | dpp4\_23 | 4.9200 | 51 | 240 | -4.8000 | moderately active | [11 14 \* 25 ] | | dpp4\_24 | 4.0500 | 380 | 440 | -1.2000 | inactive | [\* 15 \* 24 ] | | dpp4\_25 | 4.1300 | 310 | 540 | -1.7000 | inactive | [\* 6 13 19 ] | | dpp4\_26 | 4.0200 | 410 | 800 | -2 | inactive | [\* 16 \* 32 ] | | dpp4\_27 | 3.7100 | 830 | 1000 | -1.2000 | inactive | [\* \* 23 19 ] | | dpp4\_28 | 4.0500 | 380 | 1000 | -2.7000 | inactive | [\* \* 15 10 ] | |

Hypothesis 9

|  |  |  |  |  |  |  |  |  |  |  |  |  |  |  |  |  |  |  |  |  |  |  |  |  |  |  |  |  |  |  |  |  |  |  |  |  |  |  |  |  |  |  |  |  |  |  |  |  |  |  |  |  |  |  |  |  |  |  |  |  |  |  |  |  |  |  |  |  |  |  |  |  |  |  |  |  |  |  |  |  |  |  |  |  |  |  |  |  |  |  |  |  |  |  |  |  |  |  |  |  |  |  |  |  |  |  |  |  |  |  |  |  |  |  |  |  |  |  |  |  |  |  |  |  |  |  |  |  |  |  |  |  |  |  |  |  |  |  |  |  |  |  |  |  |  |  |  |  |  |  |  |  |  |  |  |  |  |  |  |  |  |  |  |  |  |  |  |  |  |  |  |  |  |  |  |  |  |  |  |  |  |  |  |  |  |  |  |  |  |  |  |  |  |  |  |  |  |  |  |  |  |  |  |
| --- | --- | --- | --- | --- | --- | --- | --- | --- | --- | --- | --- | --- | --- | --- | --- | --- | --- | --- | --- | --- | --- | --- | --- | --- | --- | --- | --- | --- | --- | --- | --- | --- | --- | --- | --- | --- | --- | --- | --- | --- | --- | --- | --- | --- | --- | --- | --- | --- | --- | --- | --- | --- | --- | --- | --- | --- | --- | --- | --- | --- | --- | --- | --- | --- | --- | --- | --- | --- | --- | --- | --- | --- | --- | --- | --- | --- | --- | --- | --- | --- | --- | --- | --- | --- | --- | --- | --- | --- | --- | --- | --- | --- | --- | --- | --- | --- | --- | --- | --- | --- | --- | --- | --- | --- | --- | --- | --- | --- | --- | --- | --- | --- | --- | --- | --- | --- | --- | --- | --- | --- | --- | --- | --- | --- | --- | --- | --- | --- | --- | --- | --- | --- | --- | --- | --- | --- | --- | --- | --- | --- | --- | --- | --- | --- | --- | --- | --- | --- | --- | --- | --- | --- | --- | --- | --- | --- | --- | --- | --- | --- | --- | --- | --- | --- | --- | --- | --- | --- | --- | --- | --- | --- | --- | --- | --- | --- | --- | --- | --- | --- | --- | --- | --- | --- | --- | --- | --- | --- | --- | --- | --- | --- | --- | --- | --- | --- | --- | --- | --- | --- | --- | --- | --- |
| |  |  |  |  |  |  |  |  |  |  |  |  |  |  |  |  |  |  |  | | --- | --- | --- | --- | --- | --- | --- | --- | --- | --- | --- | --- | --- | --- | --- | --- | --- | --- | --- | | |  |  |  |  |  |  |  |  |  |  |  |  |  |  |  |  |  |  | | --- | --- | --- | --- | --- | --- | --- | --- | --- | --- | --- | --- | --- | --- | --- | --- | --- | --- | | |  |  | | --- | --- | | Results | | | Maximum Fit | 7.96869 | | Total Cost | 192.708 | | RMS | 3.06002 | | Correlation | 0.853487 | | |  |  | | --- | --- | | Description | | | Features | Weights | | HBA  HBA\_lipid  HBD  HYDROPHOBIC | 1.99217  1.99217  1.99217  1.99217 | | | |  | | |  |  |  |  |  |  |  | | --- | --- | --- | --- | --- | --- | --- | | Name | Fit | Est | Act | Err | Status | Mapping | | dpp4\_1 | 6.7600 | 0.86000 | 0.12 | 7.1000 | active | [25 14 13 1 ] | | dpp4\_2 | 7.0500 | 0.44000 | 0.24 | 1.8000 | active | [25 17 13 1 ] | | dpp4\_4 | 5.6900 | 10 | 2 | 5.1000 | moderately active | [30 17 \* 22 ] | | dpp4\_3 | 5.8300 | 7.4000 | 2 | 3.7000 | moderately active | [18 20 21 1 ] | | dpp4\_7 | 5.8400 | 7.1000 | 5.8 | 1.2000 | moderately active | [28 \* 21 1 ] | | dpp4\_8 | 5.7600 | 8.6000 | 9.6 | -1.1000 | moderately active | [30 11 \* 23 ] | | dpp4\_9 | 5.7800 | 8.3000 | 12 | -1.4000 | moderately active | [\* 20 21 1 ] | | dpp4\_10 | 5.0800 | 41 | 16 | 2.6000 | moderately active | [\* 17 14 1 ] | | dpp4\_11 | 5.4900 | 16 | 17 | -1.1000 | moderately active | [\* 23 13 9 ] | | dpp4\_12 | 5.2600 | 27 | 43 | -1.6000 | moderately active | [\* 13 15 16 ] | | dpp4\_13 | 5.7800 | 8.3000 | 44 | -5.3000 | moderately active | [\* 17 14 1 ] | | dpp4\_14 | 4.2100 | 310 | 45 | 6.8000 | moderately active | [30 17 \* 21 ] | | dpp4\_15 | 5.2100 | 31 | 50 | -1.6000 | moderately active | [26 \* 21 1 ] | | dpp4\_17 | 3.9800 | 510 | 64 | 8 | moderately active | [\* \* 21 25 ] | | dpp4\_18 | 5.4600 | 17 | 120 | -7 | moderately active | [42 36 \* 32 ] | | dpp4\_19 | 5.4200 | 19 | 130 | -6.7000 | moderately active | [27 \* 15 10 ] | | dpp4\_20 | 4.8300 | 74 | 140 | -1.8000 | moderately active | [11 14 \* 25 ] | | dpp4\_21 | 3.8900 | 630 | 180 | 3.5000 | moderately active | [\* 12 \* 24 ] | | dpp4\_22 | 3.9700 | 530 | 220 | 2.4000 | moderately active | [\* \* 21 1 ] | | dpp4\_23 | 4.8100 | 76 | 240 | -3.2000 | moderately active | [11 14 \* 25 ] | | dpp4\_24 | 4.1300 | 370 | 440 | -1.2000 | inactive | [\* 13 15 10 ] | | dpp4\_25 | 4.6500 | 110 | 540 | -4.8000 | inactive | [\* 23 13 9 ] | | dpp4\_26 | 3.9800 | 520 | 800 | -1.5000 | inactive | [\* 21 \* 14 ] | | dpp4\_27 | 3.6800 | 1000 | 1000 | 1 | inactive | [\* 8 \* 24 ] | | dpp4\_28 | 4.4800 | 160 | 1000 | -6.3000 | inactive | [\* 22 15 10 ] | |

Hypothesis 10

|  |  |  |  |  |  |  |  |  |  |  |  |  |  |  |  |  |  |  |  |  |  |  |  |  |  |  |  |  |  |  |  |  |  |  |  |  |  |  |  |  |  |  |  |  |  |  |  |  |  |  |  |  |  |  |  |  |  |  |  |  |  |  |  |  |  |  |  |  |  |  |  |  |  |  |  |  |  |  |  |  |  |  |  |  |  |  |  |  |  |  |  |  |  |  |  |  |  |  |  |  |  |  |  |  |  |  |  |  |  |  |  |  |  |  |  |  |  |  |  |  |  |  |  |  |  |  |  |  |  |  |  |  |  |  |  |  |  |  |  |  |  |  |  |  |  |  |  |  |  |  |  |  |  |  |  |  |  |  |  |  |  |  |  |  |  |  |  |  |  |  |  |  |  |  |  |  |  |  |  |  |  |  |  |  |  |  |  |  |  |  |  |  |  |  |  |  |  |  |  |  |  |  |  |
| --- | --- | --- | --- | --- | --- | --- | --- | --- | --- | --- | --- | --- | --- | --- | --- | --- | --- | --- | --- | --- | --- | --- | --- | --- | --- | --- | --- | --- | --- | --- | --- | --- | --- | --- | --- | --- | --- | --- | --- | --- | --- | --- | --- | --- | --- | --- | --- | --- | --- | --- | --- | --- | --- | --- | --- | --- | --- | --- | --- | --- | --- | --- | --- | --- | --- | --- | --- | --- | --- | --- | --- | --- | --- | --- | --- | --- | --- | --- | --- | --- | --- | --- | --- | --- | --- | --- | --- | --- | --- | --- | --- | --- | --- | --- | --- | --- | --- | --- | --- | --- | --- | --- | --- | --- | --- | --- | --- | --- | --- | --- | --- | --- | --- | --- | --- | --- | --- | --- | --- | --- | --- | --- | --- | --- | --- | --- | --- | --- | --- | --- | --- | --- | --- | --- | --- | --- | --- | --- | --- | --- | --- | --- | --- | --- | --- | --- | --- | --- | --- | --- | --- | --- | --- | --- | --- | --- | --- | --- | --- | --- | --- | --- | --- | --- | --- | --- | --- | --- | --- | --- | --- | --- | --- | --- | --- | --- | --- | --- | --- | --- | --- | --- | --- | --- | --- | --- | --- | --- | --- | --- | --- | --- | --- | --- | --- | --- | --- | --- | --- | --- | --- | --- | --- |
| |  |  |  |  |  |  |  |  |  |  |  |  |  |  |  |  |  |  |  | | --- | --- | --- | --- | --- | --- | --- | --- | --- | --- | --- | --- | --- | --- | --- | --- | --- | --- | --- | | |  |  |  |  |  |  |  |  |  |  |  |  |  |  |  |  |  |  | | --- | --- | --- | --- | --- | --- | --- | --- | --- | --- | --- | --- | --- | --- | --- | --- | --- | --- | | |  |  | | --- | --- | | Results | | | Maximum Fit | 7.51203 | | Total Cost | 203.411 | | RMS | 3.19585 | | Correlation | 0.838927 | | |  |  | | --- | --- | | Description | | | Features | Weights | | HBA\_lipid  HBA\_lipid  HBD  HYDROPHOBIC | 1.87801  1.87801  1.87801  1.87801 | | | |  | | |  |  |  |  |  |  |  | | --- | --- | --- | --- | --- | --- | --- | | Name | Fit | Est | Act | Err | Status | Mapping | | dpp4\_1 | 6.6900 | 0.51000 | 0.12 | 4.3000 | active | [25 14 13 1 ] | | dpp4\_2 | 6.8800 | 0.33000 | 0.24 | 1.4000 | active | [25 17 13 1 ] | | dpp4\_4 | 5.3700 | 11 | 2 | 5.3000 | moderately active | [30 17 \* 22 ] | | dpp4\_3 | 5.4400 | 9.1000 | 2 | 4.6000 | moderately active | [18 20 21 1 ] | | dpp4\_7 | 5.3600 | 11 | 5.8 | 1.9000 | moderately active | [27 \* 21 1 ] | | dpp4\_8 | 5.3800 | 10 | 9.6 | 1.1000 | moderately active | [30 11 \* 23 ] | | dpp4\_9 | 5.3000 | 12 | 12 | 1 | moderately active | [\* 20 21 1 ] | | dpp4\_10 | 4.5900 | 63 | 16 | 4 | moderately active | [\* 17 14 1 ] | | dpp4\_11 | 4.7000 | 50 | 17 | 2.9000 | moderately active | [\* 23 13 9 ] | | dpp4\_12 | 4.8000 | 40 | 43 | -1.1000 | moderately active | [\* 13 15 16 ] | | dpp4\_13 | 5.4800 | 8.3000 | 44 | -5.3000 | moderately active | [\* 17 14 1 ] | | dpp4\_14 | 4.1500 | 180 | 45 | 3.9000 | moderately active | [30 17 \* 21 ] | | dpp4\_15 | 4.7000 | 50 | 50 | 1 | moderately active | [26 \* 21 1 ] | | dpp4\_17 | 3.7100 | 480 | 64 | 7.5000 | moderately active | [\* \* 21 25 ] | | dpp4\_18 | 5.2400 | 14 | 120 | -8.3000 | moderately active | [42 36 \* 32 ] | | dpp4\_19 | 5.0300 | 23 | 130 | -5.4000 | moderately active | [26 \* 15 10 ] | | dpp4\_20 | 4.8500 | 36 | 140 | -3.8000 | moderately active | [21 13 \* 29 ] | | dpp4\_21 | 3.6700 | 530 | 180 | 3 | moderately active | [\* 12 \* 24 ] | | dpp4\_22 | 3.7600 | 440 | 220 | 2 | moderately active | [\* \* 21 1 ] | | dpp4\_23 | 5 | 25 | 240 | -9.9000 | moderately active | [21 14 \* 29 ] | | dpp4\_24 | 4.1200 | 190 | 440 | -2.3000 | inactive | [\* 13 15 10 ] | | dpp4\_25 | 3.7500 | 440 | 540 | -1.2000 | inactive | [\* 13 \* 19 ] | | dpp4\_26 | 3.7500 | 440 | 800 | -1.8000 | inactive | [\* 21 \* 14 ] | | dpp4\_27 | 3.4700 | 840 | 1000 | -1.2000 | inactive | [\* 8 \* 24 ] | | dpp4\_28 | 4.4000 | 100 | 1000 | -10 | inactive | [\* 22 15 10 ] | |
