# Supplementary material for: Discovery of Novel DPP-IV Inhibitors as Potential Candidates for the Treatment of Type 2 Diabetes Mellitus Predicted by 3D QSAR Pharmacophore Models, Molecular Docking and De Novo Evolution
Source: Molecules. 2019 Aug 7;24(16):2870. doi: 10.3390/molecules24162870 (PMC6720998; doi:10.3390/molecules24162870)
Supplement: Supplementary file 1 [file molecules-24-02870-s001.zip › Supplementary materials/3DQSARPharmacophoreGeneration/Report.htm]

3D QSAR Pharmacophore Generation


|  |
| --- |
| 3D QSAR Pharmacophore Generation |
| --- |
| |  |  | | --- | --- | | Information |  |  |  |  |  |  | | --- | --- | --- | --- | | |  |  |  | | --- | --- | --- | |  | Status | Success | | | |  |  |  | | --- | --- | --- | |  | User | thtf | | | |  |  |  | | --- | --- | --- | |  | Elapsed Time | 02:37:37 | | | |  |  | | --- | --- | | Start | 05/13/19 04:35:34 | | Finish | 05/13/19 07:13:11 | | | |  |  |  | | --- | --- | --- | |  | Server Name | harvey-peter | | | |  |  | | --- | --- | | DS Version | 18.1.100.18066 | | PP Version | 18.1.100.11 | | DS Client Version | 18.1.0.17334 | | Server Ports | 9944 (9943) | | OS Distribution | Redhat | | OS Version | 6.5 | | Memory Total | 31.32 GB | | Memory Free | 27.07 GB | | |
| |  |  | | --- | --- | | Summary |  |  |  |  |  |  |  |  |  |  |  |  |  |  |  |  |  |  |  |  |  |  |  |  |  |  |  |  |  |  |  |  |  |  |  |  |  |  |  |  |  |  |  |  |  |  |  |  |  |  |  |  |  |  |  |  |  |  |  |  |  |  |  |  |  |  |  |  |  |  |  |  |  |  |  |  |  |  |  |  |  |  |  |  |  |  |  |  |  |  |  |  |  |  |  |  |  |  |  |  |  |  |  |  |  |  |  |  |  |  |  |  |  |  |  |  |  |  |  |  |  |  |  |  |  |  |  |  |  |  |  |  |  |  |  |  |  |  |  |  |  |  |  |  |  |  |  |  |  |  |  |  |  |  |  |  |  |  |  |  |  |  |  |  |  |  |  |  | | --- | --- | --- | --- | --- | --- | --- | --- | --- | --- | --- | --- | --- | --- | --- | --- | --- | --- | --- | --- | --- | --- | --- | --- | --- | --- | --- | --- | --- | --- | --- | --- | --- | --- | --- | --- | --- | --- | --- | --- | --- | --- | --- | --- | --- | --- | --- | --- | --- | --- | --- | --- | --- | --- | --- | --- | --- | --- | --- | --- | --- | --- | --- | --- | --- | --- | --- | --- | --- | --- | --- | --- | --- | --- | --- | --- | --- | --- | --- | --- | --- | --- | --- | --- | --- | --- | --- | --- | --- | --- | --- | --- | --- | --- | --- | --- | --- | --- | --- | --- | --- | --- | --- | --- | --- | --- | --- | --- | --- | --- | --- | --- | --- | --- | --- | --- | --- | --- | --- | --- | --- | --- | --- | --- | --- | --- | --- | --- | --- | --- | --- | --- | --- | --- | --- | --- | --- | --- | --- | --- | --- | --- | --- | --- | --- | --- | --- | --- | --- | --- | --- | --- | --- | --- | --- | --- | --- | --- | --- | --- | --- | --- | --- | --- | --- | --- | | |  |  |  |  |  |  |  |  |  |  |  |  |  | | --- | --- | --- | --- | --- | --- | --- | --- | --- | --- | --- | --- | --- | | |  | | --- | | Confidence Level - 95% | | Significance for hypothesis 1 is 95% | | Significance for hypothesis 2 is 95% | | Significance for hypothesis 3 is 95% | | Significance for hypothesis 4 is 95% | | Significance for hypothesis 5 is 95% | | Significance for hypothesis 6 is 95% | | Significance for hypothesis 7 is 95% | | Significance for hypothesis 8 is 95% | | Significance for hypothesis 9 is 95% | | Significance for hypothesis 10 is 95% | | Generated 10 hypotheses | | | |  |  |  |  |  |  |  |  |  |  |  |  |  |  |  |  |  |  |  |  |  |  |  |  |  |  |  |  |  |  |  |  |  |  |  |  |  |  |  |  |  |  |  |  |  |  |  |  |  |  |  |  |  |  |  |  |  |  |  |  |  |  |  |  |  |  |  |  |  |  |  |  |  |  |  |  |  |  |  |  |  |  |  |  |  |  |  |  |  |  |  |  |  |  |  |  |  |  |  |  |  |  |  |  |  |  |  |  |  |  |  |  |  |  |  |  |  |  |  |  |  |  |  |  |  |  |  |  |  |  |  |  |  |  |  |  |  |  |  |  |  |  |  |  |  |  |  |  |  |  |  | | --- | --- | --- | --- | --- | --- | --- | --- | --- | --- | --- | --- | --- | --- | --- | --- | --- | --- | --- | --- | --- | --- | --- | --- | --- | --- | --- | --- | --- | --- | --- | --- | --- | --- | --- | --- | --- | --- | --- | --- | --- | --- | --- | --- | --- | --- | --- | --- | --- | --- | --- | --- | --- | --- | --- | --- | --- | --- | --- | --- | --- | --- | --- | --- | --- | --- | --- | --- | --- | --- | --- | --- | --- | --- | --- | --- | --- | --- | --- | --- | --- | --- | --- | --- | --- | --- | --- | --- | --- | --- | --- | --- | --- | --- | --- | --- | --- | --- | --- | --- | --- | --- | --- | --- | --- | --- | --- | --- | --- | --- | --- | --- | --- | --- | --- | --- | --- | --- | --- | --- | --- | --- | --- | --- | --- | --- | --- | --- | --- | --- | --- | --- | --- | --- | --- | --- | --- | --- | --- | --- | --- | --- | --- | --- | --- | --- | --- | --- | --- | --- | --- | | |  |  |  |  |  |  |  |  |  |  |  |  |  |  |  |  |  |  |  |  |  |  |  |  |  |  |  |  |  |  | | --- | --- | --- | --- | --- | --- | --- | --- | --- | --- | --- | --- | --- | --- | --- | --- | --- | --- | --- | --- | --- | --- | --- | --- | --- | --- | --- | --- | --- | --- | | |  |  |  |  |  |  |  |  |  |  |  |  |  | | --- | --- | --- | --- | --- | --- | --- | --- | --- | --- | --- | --- | --- | | |  |  |  |  | | --- | --- | --- | --- | | Validation Result Using Input Test Ligands | | | | | Model Name | q^2 | RMS Error | Mean Absolute Error | | training\_set-(1)\_01.chm | 0.438 | 0.673 | 0.510 | | |  | | |  |  |  |  |  |  |  |  |  |  |  |  |  | | --- | --- | --- | --- | --- | --- | --- | --- | --- | --- | --- | --- | --- | | |  |  |  |  | | --- | --- | --- | --- | | Validation Result Using Input Test Ligands | | | | | Model Name | q^2 | RMS Error | Mean Absolute Error | | training\_set-(1)\_02.chm | 0.665 | 0.532 | 0.411 | | |  | | | |  |  |  |  |  |  |  |  |  |  |  |  |  | | --- | --- | --- | --- | --- | --- | --- | --- | --- | --- | --- | --- | --- | | |  |  |  |  | | --- | --- | --- | --- | | Validation Result Using Input Test Ligands | | | | | Model Name | q^2 | RMS Error | Mean Absolute Error | | training\_set-(1)\_03.chm | 0.480 | 0.658 | 0.555 | | |  | | |  |  |  |  |  |  |  |  |  |  |  |  |  | | --- | --- | --- | --- | --- | --- | --- | --- | --- | --- | --- | --- | --- | | |  |  |  |  | | --- | --- | --- | --- | | Validation Result Using Input Test Ligands | | | | | Model Name | q^2 | RMS Error | Mean Absolute Error | | training\_set-(1)\_04.chm | 0.517 | 0.617 | 0.490 | | |  | | | |  |  |  |  |  |  |  |  |  |  |  |  |  | | --- | --- | --- | --- | --- | --- | --- | --- | --- | --- | --- | --- | --- | | |  |  |  |  | | --- | --- | --- | --- | | Validation Result Using Input Test Ligands | | | | | Model Name | q^2 | RMS Error | Mean Absolute Error | | training\_set-(1)\_05.chm | 0.351 | 0.743 | 0.625 | | |  | | |  |  |  |  |  |  |  |  |  |  |  |  |  | | --- | --- | --- | --- | --- | --- | --- | --- | --- | --- | --- | --- | --- | | |  |  |  |  | | --- | --- | --- | --- | | Validation Result Using Input Test Ligands | | | | | Model Name | q^2 | RMS Error | Mean Absolute Error | | training\_set-(1)\_06.chm | 0.563 | 0.693 | 0.559 | | |  | | | |  |  |  |  |  |  |  |  |  |  |  |  |  | | --- | --- | --- | --- | --- | --- | --- | --- | --- | --- | --- | --- | --- | | |  |  |  |  | | --- | --- | --- | --- | | Validation Result Using Input Test Ligands | | | | | Model Name | q^2 | RMS Error | Mean Absolute Error | | training\_set-(1)\_07.chm | 0.466 | 0.656 | 0.503 | | |  | | |  |  |  |  |  |  |  |  |  |  |  |  |  | | --- | --- | --- | --- | --- | --- | --- | --- | --- | --- | --- | --- | --- | | |  |  |  |  | | --- | --- | --- | --- | | Validation Result Using Input Test Ligands | | | | | Model Name | q^2 | RMS Error | Mean Absolute Error | | training\_set-(1)\_08.chm | 0.414 | 0.754 | 0.607 | | |  | | | |  |  |  |  |  |  |  |  |  |  |  |  |  | | --- | --- | --- | --- | --- | --- | --- | --- | --- | --- | --- | --- | --- | | |  |  |  |  | | --- | --- | --- | --- | | Validation Result Using Input Test Ligands | | | | | Model Name | q^2 | RMS Error | Mean Absolute Error | | training\_set-(1)\_09.chm | 0.623 | 0.558 | 0.425 | | |  | | |  |  |  |  |  |  |  |  |  |  |  |  |  | | --- | --- | --- | --- | --- | --- | --- | --- | --- | --- | --- | --- | --- | | |  |  |  |  | | --- | --- | --- | --- | | Validation Result Using Input Test Ligands | | | | | Model Name | q^2 | RMS Error | Mean Absolute Error | | training\_set-(1)\_10.chm | 0.679 | 0.546 | 0.444 | | |  | | | | | |
| |  |  | | --- | --- | | Details |  |  |  |  |  |  |  |  |  |  |  |  |  |  |  |  |  |  |  |  |  |  |  |  |  |  |  |  |  |  |  |  |  |  |  |  |  |  |  |  |  |  |  |  |  |  |  |  |  |  |  |  |  |  |  |  |  |  |  |  |  |  |  |  |  |  |  |  |  |  |  |  |  |  |  |  |  |  |  |  |  |  |  |  |  |  |  |  |  |  |  |  |  |  |  |  |  |  |  |  |  | | --- | --- | --- | --- | --- | --- | --- | --- | --- | --- | --- | --- | --- | --- | --- | --- | --- | --- | --- | --- | --- | --- | --- | --- | --- | --- | --- | --- | --- | --- | --- | --- | --- | --- | --- | --- | --- | --- | --- | --- | --- | --- | --- | --- | --- | --- | --- | --- | --- | --- | --- | --- | --- | --- | --- | --- | --- | --- | --- | --- | --- | --- | --- | --- | --- | --- | --- | --- | --- | --- | --- | --- | --- | --- | --- | --- | --- | --- | --- | --- | --- | --- | --- | --- | --- | --- | --- | --- | --- | --- | --- | --- | --- | --- | --- | --- | --- | --- | --- | | |  |  |  |  |  |  |  |  |  |  |  |  |  |  |  |  |  |  |  |  |  |  |  |  |  |  |  |  |  |  |  |  |  |  |  |  |  |  |  |  |  |  |  |  |  |  |  |  |  |  |  |  |  |  |  |  |  |  |  |  |  |  |  |  |  |  |  |  |  |  |  |  |  |  |  |  |  |  |  |  |  |  |  |  |  |  |  |  |  |  |  |  |  |  |  |  |  |  | | --- | --- | --- | --- | --- | --- | --- | --- | --- | --- | --- | --- | --- | --- | --- | --- | --- | --- | --- | --- | --- | --- | --- | --- | --- | --- | --- | --- | --- | --- | --- | --- | --- | --- | --- | --- | --- | --- | --- | --- | --- | --- | --- | --- | --- | --- | --- | --- | --- | --- | --- | --- | --- | --- | --- | --- | --- | --- | --- | --- | --- | --- | --- | --- | --- | --- | --- | --- | --- | --- | --- | --- | --- | --- | --- | --- | --- | --- | --- | --- | --- | --- | --- | --- | --- | --- | --- | --- | --- | --- | --- | --- | --- | --- | --- | --- | --- | --- | | |  | | --- | | Scramble run 1 created 10 valid hypotheses and will be included in data tables | | Scramble run 2 created 10 valid hypotheses and will be included in data tables | | Scramble run 3 created 10 valid hypotheses and will be included in data tables | | Scramble run 4 created 10 valid hypotheses and will be included in data tables | | Scramble run 5 created 10 valid hypotheses and will be included in data tables | | Scramble run 6 created 10 valid hypotheses and will be included in data tables | | Scramble run 7 created 10 valid hypotheses and will be included in data tables | | Scramble run 8 created 10 valid hypotheses and will be included in data tables | | Scramble run 9 created 10 valid hypotheses and will be included in data tables | | Scramble run 10 created 10 valid hypotheses and will be included in data tables | | Scramble run 11 created 10 valid hypotheses and will be included in data tables | | Scramble run 12 created 10 valid hypotheses and will be included in data tables | | Scramble run 13 created 10 valid hypotheses and will be included in data tables | | Scramble run 14 created 10 valid hypotheses and will be included in data tables | | Scramble run 15 created 10 valid hypotheses and will be included in data tables | | Scramble run 16 created 10 valid hypotheses and will be included in data tables | | Scramble run 17 created 10 valid hypotheses and will be included in data tables | | Scramble run 18 created 10 valid hypotheses and will be included in data tables | | Scramble run 19 created 10 valid hypotheses and will be included in data tables | | Hypothesis 1 | | Definition: HBA HBA\_lipid HBD HYDROPHOBIC | | Weights: 2.09701 2.09701 2.09701 2.09701 | | Tolerances: 1.60 2.20 1.60 2.20 1.60 2.20 1.60 | | totalcost=138.152 RMS=2.23498 correl=0.924736 | | Cost components: Error=121.61 Weight=1.17659 Config=15.3653 Tolerance=0 | |  | | Hypothesis 2 | | Definition: HBA HBA\_lipid HBD HYDROPHOBIC | | Weights: 2.10766 2.10766 2.10766 2.10766 | | Tolerances: 1.60 2.20 1.60 2.20 1.60 2.20 1.60 | | totalcost=139.866 RMS=2.26525 correl=0.922598 | | Cost components: Error=123.313 Weight=1.18853 Config=15.3653 Tolerance=0 | |  | | Hypothesis 3 | | Definition: HBA\_lipid HBA\_lipid HBD HYDROPHOBIC | | Weights: 2.07584 2.07584 2.07584 2.07584 | | Tolerances: 1.60 2.20 1.60 2.20 1.60 2.20 1.60 | | totalcost=164.511 RMS=2.66561 correl=0.891029 | | Cost components: Error=147.99 Weight=1.15652 Config=15.3653 Tolerance=0 | |  | | Hypothesis 4 | | Definition: HBA\_lipid HBA\_lipid HBD HYDROPHOBIC | | Weights: 2.11051 2.11051 2.11051 2.11051 | | Tolerances: 1.60 2.20 1.60 2.20 1.60 2.20 1.60 | | totalcost=167.104 RMS=2.70372 correl=0.887694 | | Cost components: Error=150.547 Weight=1.19194 Config=15.3653 Tolerance=0 | |  | | Hypothesis 5 | | Definition: HBA HBA\_lipid HBA\_lipid HYDROPHOBIC | | Weights: 2.27556 2.27556 2.27556 2.27556 | | Tolerances: 1.60 2.20 1.60 2.20 1.60 2.20 1.60 | | totalcost=174.539 RMS=2.80659 correl=0.878395 | | Cost components: Error=157.633 Weight=1.54128 Config=15.3653 Tolerance=0 | |  | | Hypothesis 6 | | Definition: HBA HBA\_lipid HBD HYDROPHOBIC | | Weights: 1.93712 1.93712 1.93712 1.93712 | | Tolerances: 1.60 2.20 1.60 2.20 1.60 2.20 1.60 | | totalcost=176.215 RMS=2.83594 correl=0.875645 | | Cost components: Error=159.703 Weight=1.14667 Config=15.3653 Tolerance=0 | |  | | Hypothesis 7 | | Definition: HBA HBA\_lipid HBD HYDROPHOBIC | | Weights: 1.83525 1.83525 1.83525 1.83525 | | Tolerances: 1.60 2.20 1.60 2.20 1.60 2.20 1.60 | | totalcost=178.056 RMS=2.86002 correl=0.873374 | | Cost components: Error=161.417 Weight=1.27379 Config=15.3653 Tolerance=0 | |  | | Hypothesis 8 | | Definition: HBA HBA\_lipid HBD HYDROPHOBIC | | Weights: 2.02312 2.02312 2.02312 2.02312 | | Tolerances: 1.60 2.20 1.60 2.20 1.60 2.20 1.60 | | totalcost=180.027 RMS=2.88947 correl=0.870556 | | Cost components: Error=163.534 Weight=1.12793 Config=15.3653 Tolerance=0 | |  | | Hypothesis 9 | | Definition: HBA HBA\_lipid HBD HYDROPHOBIC | | Weights: 1.99217 1.99217 1.99217 1.99217 | | Tolerances: 1.60 2.20 1.60 2.20 1.60 2.20 1.60 | | totalcost=192.708 RMS=3.06002 correl=0.853487 | | Cost components: Error=176.217 Weight=1.12533 Config=15.3653 Tolerance=0 | |  | | Hypothesis 10 | | Definition: HBA\_lipid HBA\_lipid HBD HYDROPHOBIC | | Weights: 1.87801 1.87801 1.87801 1.87801 | | Tolerances: 1.60 2.20 1.60 2.20 1.60 2.20 1.60 | | totalcost=203.411 RMS=3.19585 correl=0.838927 | | Cost components: Error=186.839 Weight=1.20658 Config=15.3653 Tolerance=0 | |  | | NULL Hypothesis | | totalcost=490.185 RMS=5.87206 correl=0 | | Cost components: Error=490.185 Weight=0 Config=0 Mapping=0 Tolerance=0 | |  | | Fixed Cost: | | totalcost=75.6612 RMS=0 correl=0 | | Cost components: Error=59.1709 Weight=1.12499 Config=15.3653 Tolerance=0 | |  | | | |
| |  |  | | --- | --- | | Results |  |  |  | | --- | | ActiveMolConfs.sd | | Atom Mappings | | Detailed Analysis Report | | Detailed Log File | | Ligands Aligned to Pharmacophore 01 | | Ligands Aligned to Pharmacophore 02 | | Ligands Aligned to Pharmacophore 03 | | Ligands Aligned to Pharmacophore 04 | | Ligands Aligned to Pharmacophore 05 | | Ligands Aligned to Pharmacophore 06 | | Ligands Aligned to Pharmacophore 07 | | Ligands Aligned to Pharmacophore 08 | | Ligands Aligned to Pharmacophore 09 | | Ligands Aligned to Pharmacophore 10 | | Log File | | Molecular Properties | | Output Pharmacophore 01 | | Output Pharmacophore 02 | | Output Pharmacophore 03 | | Output Pharmacophore 04 | | Output Pharmacophore 05 | | Output Pharmacophore 06 | | Output Pharmacophore 07 | | Output Pharmacophore 08 | | Output Pharmacophore 09 | | Output Pharmacophore 10 | | View Aligned Ligands 01 | | View Aligned Ligands 02 | | View Aligned Ligands 03 | | View Aligned Ligands 04 | | View Aligned Ligands 05 | | View Aligned Ligands 06 | | View Aligned Ligands 07 | | View Aligned Ligands 08 | | View Aligned Ligands 09 | | View Aligned Ligands 10 | | correlation.csv | | cost.csv | |
| |  |  | | --- | --- | | Parameters |  |  |  |  |  | | --- | --- | --- | | |  |  | | --- | --- | | Protocol Settings | Protocol.pr\_xml | | | |  |  |  |  | | --- | --- | --- | --- | |  | Input Ligands | |  | | --- | | training\_set-(1).sd | | | | |  |  | | --- | --- | | Activity Property | Activ | | Uncertainty Property | Uncert | | Logarithmic Activity | False | | Rescale Activity | False | | | |  |  |  | | --- | --- | --- | |  | Conformation Generation | FAST | | | |  |  | | --- | --- | | Maximum Conformations | 255 | | Discard Existing Conformations | True | | Energy Threshold | 20.0 | | Ring Fragments File |  | | Save Conformations | False | | Parallel Processing | False | | Parallel Processing Batch Size | 4 | | Parallel Processing Server | localhost | | Parallel Processing Server Processes | 2 | | Parallel Processing Preserve Order | True | | | |  |  |  | | --- | --- | --- | |  | Features | HB\_ACCEPTOR 0 5,HB\_ACCEPTOR\_lipid 0 5,HB\_DONOR 0 5,HYDROPHOBIC 0 5,HYDROPHOBIC\_aromatic 0 5 | | | |  |  |  | | --- | --- | --- | |  | Maximum Pharmacophores | 10 | | | |  |  |  | | --- | --- | --- | |  | Minimum Interfeature Distance | 1.5 | | | |  |  |  | | --- | --- | --- | |  | Maximum Excluded Volumes | 0 | | | |  |  |  | | --- | --- | --- | |  | Validation | True | | | |  |  |  | | --- | --- | --- | | Input Test Ligands | |  | | --- | | test\_set-(1).sd | | | Fitting Method | Rigid | | Maximum Omitted Features | -1 | | | |  |  |  | | --- | --- | --- | |  | Advanced |  | | | |  |  | | --- | --- | | Minimum Features | 1 | | Maximum Features | 5 | | Minimum Feature Points | 4 | | Minimum Subset Points | 4 | | Weight Variation | 0.302 | | Variable Weights | False | | Variable Tolerances | False | | Scale Feature Blob Size | 1.0 | | Inactive Spread | 3.5 | | Explore Exhaustive HBond Geometry | True | | Align Ligands to Hypothesis | True | | Best Mapping Only | True | | Fisher Validation | 95% | | Browse | False | | Catalyst Parameter File |  | | |
| ---  Copyright © 2019 Dassault Systèmes Biovia Corp - All rights reserved. |
