# Supplementary material for: Discovery of Novel DPP-IV Inhibitors as Potential Candidates for the Treatment of Type 2 Diabetes Mellitus Predicted by 3D QSAR Pharmacophore Models, Molecular Docking and De Novo Evolution
Source: Molecules. 2019 Aug 7;24(16):2870. doi: 10.3390/molecules24162870 (PMC6720998; doi:10.3390/molecules24162870)
Supplement: Supplementary file 1 [file molecules-24-02870-s001.zip › Supplementary materials/Supplementary materials-final.pdf]

molecules

MDPI

SUPPLEMENTARY MATERIALS

Discovery of Novel DPP-IV Inhibitors as Potential Candidates for the Treatment of Type 2 Diabetes Mellitus predicted by 3D QSAR pharmacophore models, molecular docking and de novo evolution

Azizullo Musoev[1,2], Sodik Numonov[1,3], Zhuhong You[1], Hongwei Gao[4],*

[1]Key Laboratory of Plant Resources and Chemistry in Arid Regions, Xinjiang Technical Institute of Physics and Chemistry, Chinese Academy of Sciences, Urumqi 830011, China; e-mail@e-azizullo.dr@gmail.com (A.M.); zhuhongyou@ms.xjb.ac.cn (Zh.Y.)

[2]University of Chinese Academy of Sciences, Beijing, 100190, China

[3]Research Institution "Chinese-Tajik Innovation Center for Natural Products" 734063, Dushanbe, Tajikistan; sodikjon82@gmail.com (S.N.)

[4]School of Life Science, Ludong University, Yantai 264025, China; gaohongw369@ms.xjb.ac.cn (H.G.)

* Corresponding author: Professor, Hongwei Gao, E-mail: gaohongw369@ms.xjb.ac.cn

Table S1: Results of the top ten pharmacophore models calculated by the HypoGen program.

Hypo No.	Total cost	Null cost Distance	Error	RMS	Correlation	Features
1	138.152	352.03	121.61	2.234	0.925	HBA, HBA_l, HBD, HY
2	139.866	350.32	123.313	2.265	0.922	HBA, HBA_l, HBD, HY
3	164.511	325.67	147.99	2.665	0.891	HBA_l, HBA_l, HBD, HY
4	167.104	323.08	150.547	2.703	0.887	HBA_l, HBA_l, HBD, HY
5	174.539	315.65	157.633	2.806	0.878	HBA, HBA_l, HBA_l, HY
6	176.215	313.97	159.703	2.835	0.875	HBA, HBA_l, HBD, HY
7	178.056	312.13	161.417	2.860	0.873	HBA, HBA_l, HBD, HY
8	180.027	310.16	163.534	2.889	0.870	HBA, HBA_l, HBD, HY
9	192.708	297.48	176.217	3.060	0.853	HBA, HBA_l, HBD, HY
10	203.411	286.77	186.839	3.195	0.838	HBA_l, HBA_l, HBD, HY

Null cost=490.185, fixed cost=75.661, for the hypo1 weight=1.176,

molecules MDPI

configuration=15.365. HBA， HBA_l, HBD， HY and HYAr are represented as hydrogen bond acceptor, hydrogen bond acceptor lipid, hydrogen bond donor, hydrophobic, and hydrophobic aromatic, respectively.

Table S2: The interacting amino acids in the ligand-protein complex for the top 11 docking compounds

Rank	Compound	Interaction amino acids
1	dpp4_42	**ARG125, GLU205, GLU206**, **TYR662,** ASP663**, TYR666,** ARG669
2	dpp4_43	**ARG125, GLU205, GLU206**, SER630, TYR631, VAL656, TRP659, **TYR662, TYR666,** VAL711, HIS740
3	dpp4_44	**ARG125**, **GLU206**, PHE357, **TYR547**, **TYR662, TYR666**
4	dpp4_45	**ARG125, GLU206, TYR547**, **TYR662**
5	dpp4_46	**GLU205,** SER209, **TYR662, TYR666,** ASN710**,** HIS740
6	dpp4_47	**ARG125**, **GLU206**, VAL207, **TYR547, TYR662, TYR666,** HIS740
7	dpp4_48	SER630**,** TYR631, VAL656, TRP659, **TYR662, TYR666,** ARG669, VAL711, HIS740
8	dpp4_49	**GLU206**, PHE357, **TYR547**, **TYR662, TYR666**
9	dpp4_50	**GLU205, GLU206**, LYS554, **TYR547,** SER630, ASP663
10	dpp4_51	**ARG125, GLU205, GLU206**, PHE357, SER630, TYR631, **TYR662, TYR666**, HIS740
11	Alogliptin*	**ARG125, GLU205,** PHE357, **TYR547,** TRP629, SER630, TYR631, **TYR662, TYR666**

* represents the control compound.

molecules

MDPI

Supplemental figure captions

Figure S1: Chemical structures and biological activity of the DPP-IV inhibitors in the training set.

Figure S2: Chemical structures and biological activity of the DPP-IV inhibitors in the test set.

Figure S3. Flow chart scheme of the total experimental procedures.

molecules

MDPI

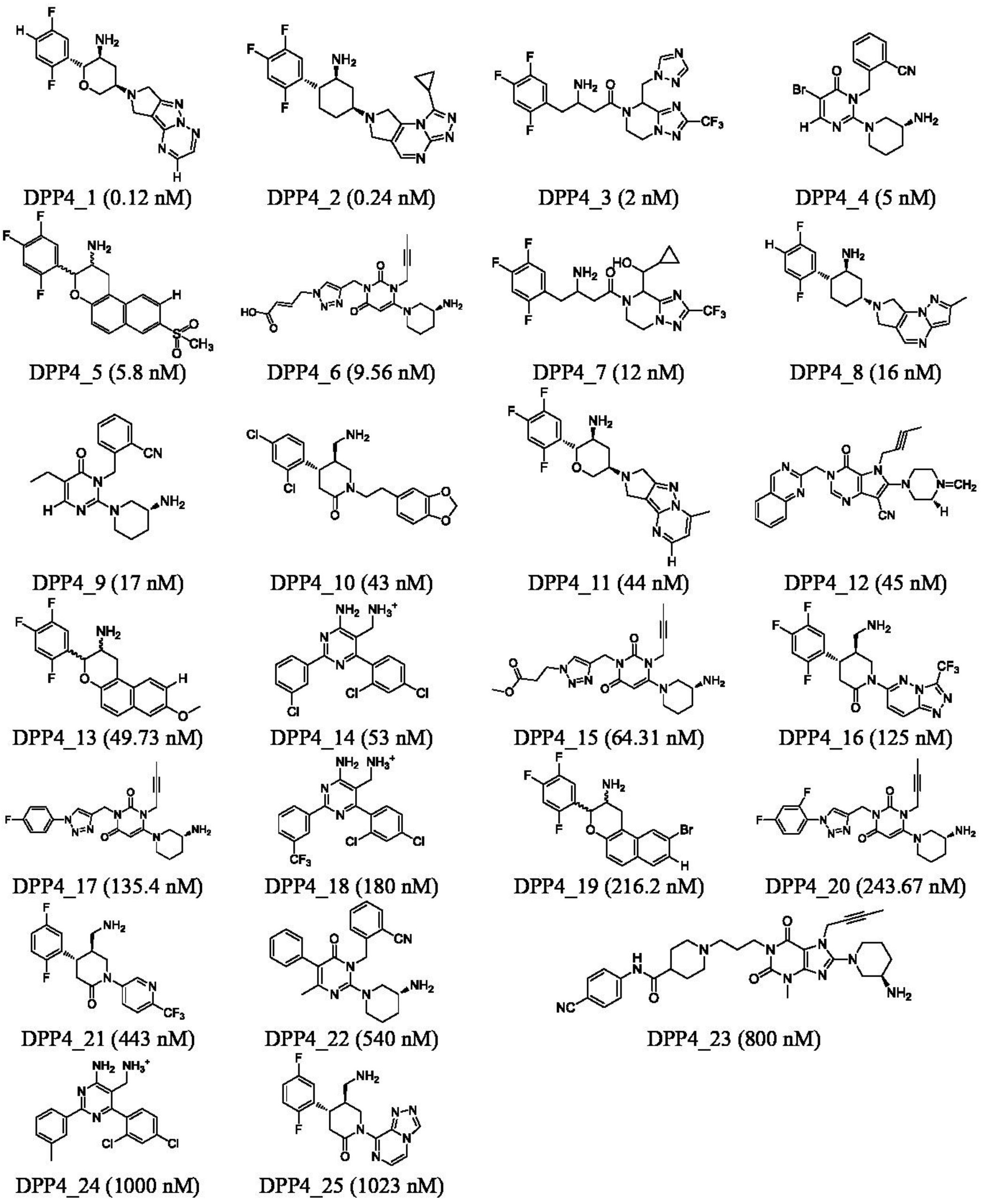

Figure S1

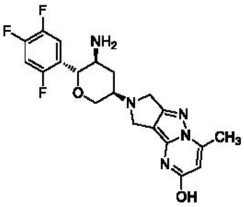

DPP4_26 (0.6 nM)

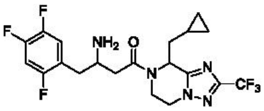

DPP4_27 (7 nM)

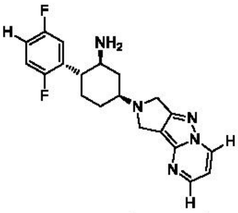

DPP4_28 (7.3 nM)

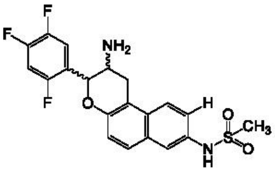

DPP4_29 (8.91 nM)

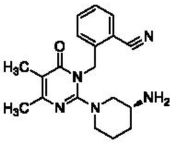

DPP4_30 (10 nM)

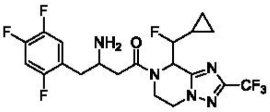

DPP4_31 (18 nM)

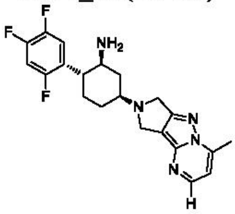

DPP4_32 (54 nM)

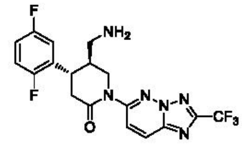

DPP4_33 (74 nM)

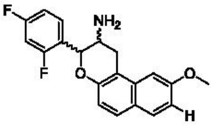

DPP4_34 (78.33 nM)

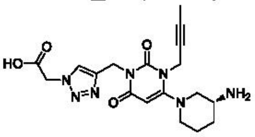

DPP4_35 (84.72 nM)

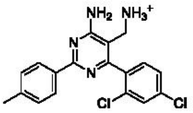

DPP4_36 (90 nM)

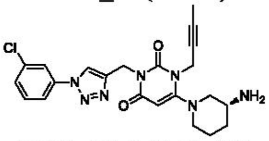

DPP4_37 (168.63 nM)

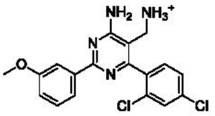

DPP4_38 (340 nM)

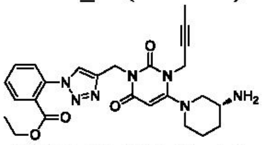

DPP4_39 (345.32 nM)

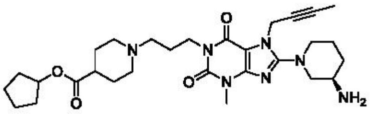

DPP4_40 (890 nM)

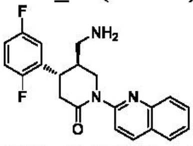

DPP4_41 (1034 nM)

Figure S2

molecules
MDPI

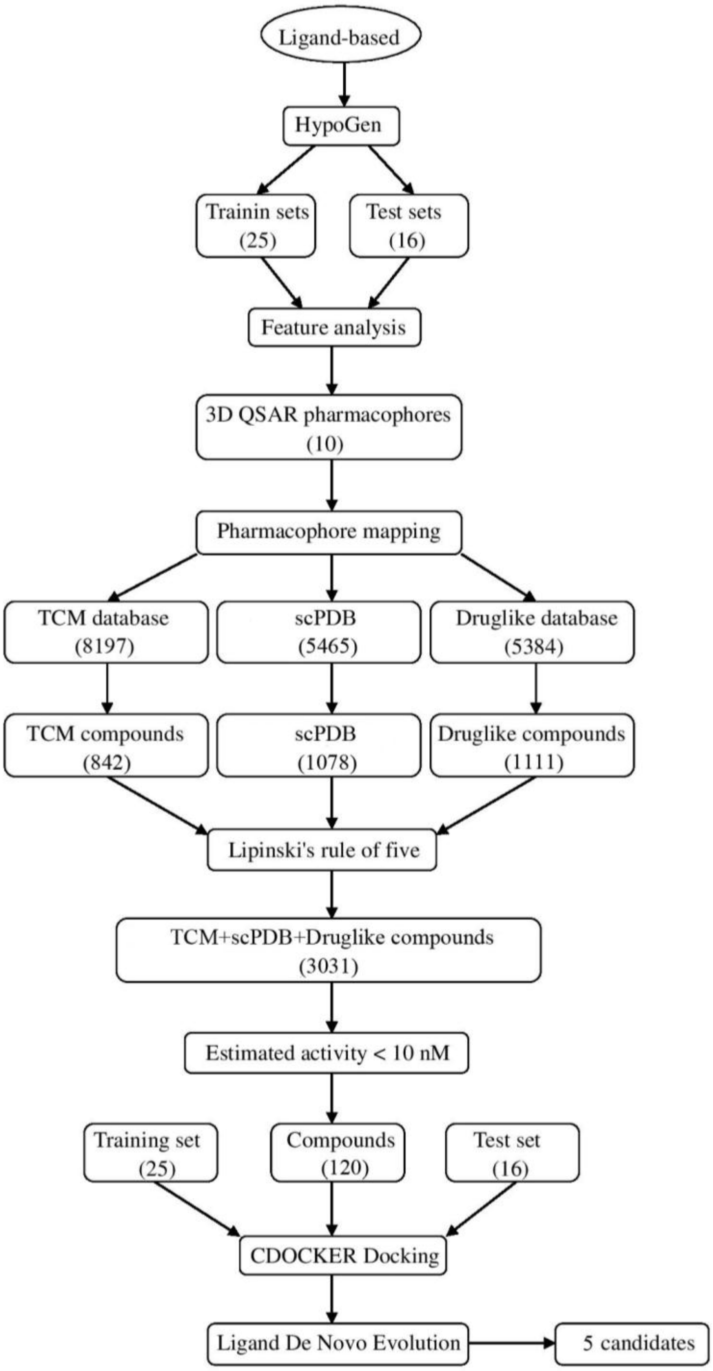

Figure S3
