# Supplementary figures and images for: Discovery of Novel DPP-IV Inhibitors as Potential Candidates for the Treatment of Type 2 Diabetes Mellitus Predicted by 3D QSAR Pharmacophore Models, Molecular Docking and De Novo Evolution
Source: Molecules. 2019 Aug 7;24(16):2870. doi: 10.3390/molecules24162870 (PMC6720998; doi:10.3390/molecules24162870)

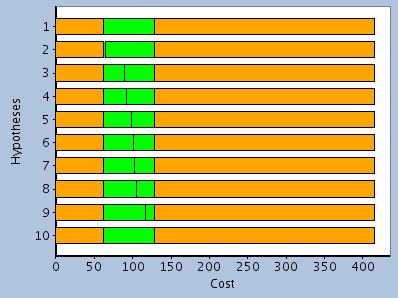

Supplement: Supplementary file 1 [file molecules-24-02870-s001.zip › Supplementary materials/3DQSARPharmacophoreGeneration/JobSummary/images/image2518723497671190165.png]

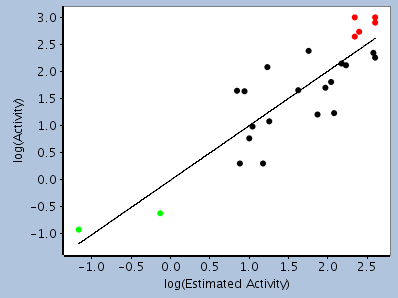

Supplement: Supplementary file 1 [file molecules-24-02870-s001.zip › Supplementary materials/3DQSARPharmacophoreGeneration/JobSummary/images/image2733613881160003228.png]

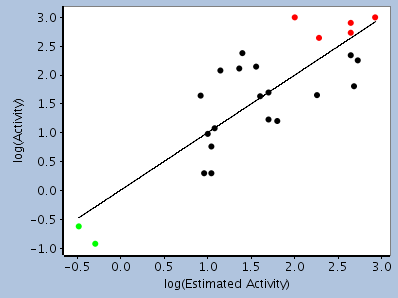

Supplement: Supplementary file 1 [file molecules-24-02870-s001.zip › Supplementary materials/3DQSARPharmacophoreGeneration/JobSummary/images/image3437834767311085730.png]

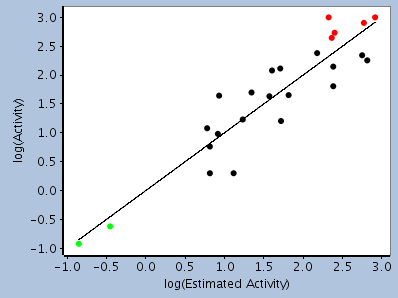

Supplement: Supplementary file 1 [file molecules-24-02870-s001.zip › Supplementary materials/3DQSARPharmacophoreGeneration/JobSummary/images/image4054887721395769746.png]

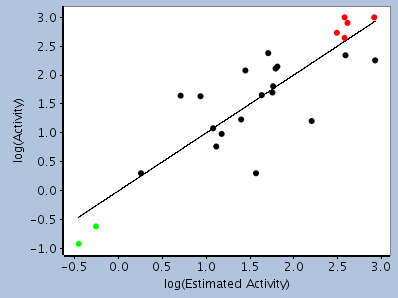

Supplement: Supplementary file 1 [file molecules-24-02870-s001.zip › Supplementary materials/3DQSARPharmacophoreGeneration/JobSummary/images/image5243565390707680346.png]

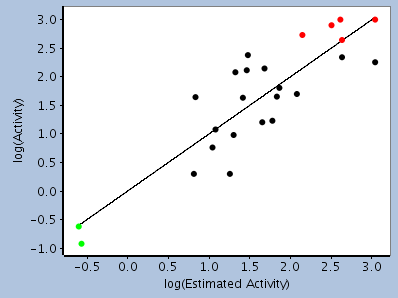

Supplement: Supplementary file 1 [file molecules-24-02870-s001.zip › Supplementary materials/3DQSARPharmacophoreGeneration/JobSummary/images/image5702412489146542867.png]

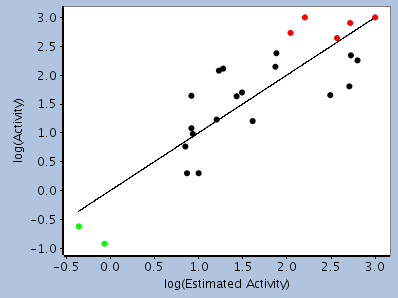

Supplement: Supplementary file 1 [file molecules-24-02870-s001.zip › Supplementary materials/3DQSARPharmacophoreGeneration/JobSummary/images/image5839113627451793708.png]

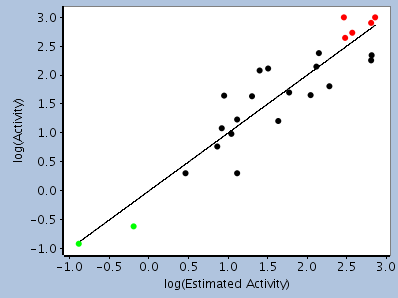

Supplement: Supplementary file 1 [file molecules-24-02870-s001.zip › Supplementary materials/3DQSARPharmacophoreGeneration/JobSummary/images/image6339569907673186466.png]

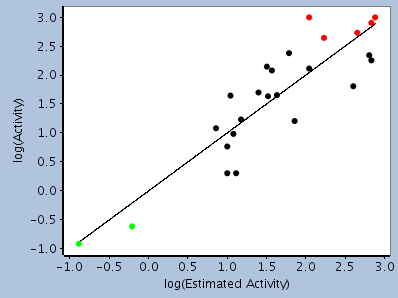

Supplement: Supplementary file 1 [file molecules-24-02870-s001.zip › Supplementary materials/3DQSARPharmacophoreGeneration/JobSummary/images/image6891303589398402374.png]

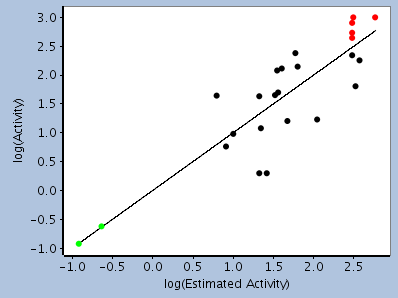

Supplement: Supplementary file 1 [file molecules-24-02870-s001.zip › Supplementary materials/3DQSARPharmacophoreGeneration/JobSummary/images/image8094802473182521902.png]

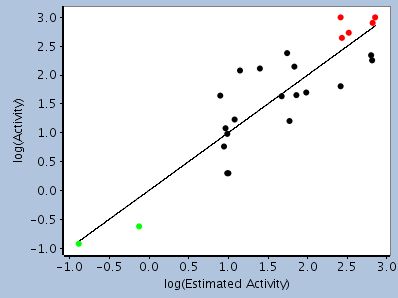

Supplement: Supplementary file 1 [file molecules-24-02870-s001.zip › Supplementary materials/3DQSARPharmacophoreGeneration/JobSummary/images/image8608152358420405832.png]

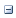

Supplement: Supplementary file 1 [file molecules-24-02870-s001.zip › Supplementary materials/3DQSARPharmacophoreGeneration/Report/images/icons/minus.png]

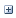

Supplement: Supplementary file 1 [file molecules-24-02870-s001.zip › Supplementary materials/3DQSARPharmacophoreGeneration/Report/images/icons/plus.png]

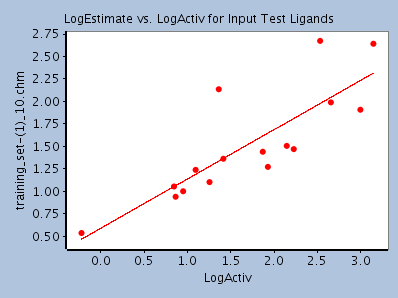

Supplement: Supplementary file 1 [file molecules-24-02870-s001.zip › Supplementary materials/3DQSARPharmacophoreGeneration/Report/images/image1153395499178645608.png]

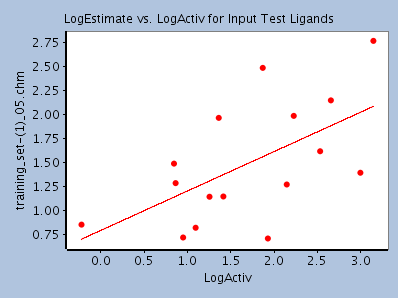

Supplement: Supplementary file 1 [file molecules-24-02870-s001.zip › Supplementary materials/3DQSARPharmacophoreGeneration/Report/images/image2201190665912450576.png]

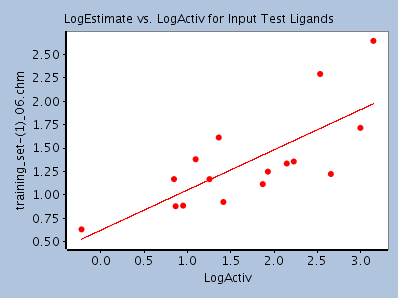

Supplement: Supplementary file 1 [file molecules-24-02870-s001.zip › Supplementary materials/3DQSARPharmacophoreGeneration/Report/images/image2582688091544882957.png]

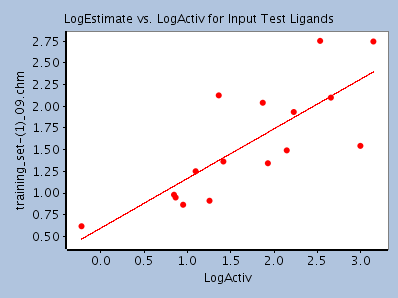

Supplement: Supplementary file 1 [file molecules-24-02870-s001.zip › Supplementary materials/3DQSARPharmacophoreGeneration/Report/images/image4962472287055113100.png]

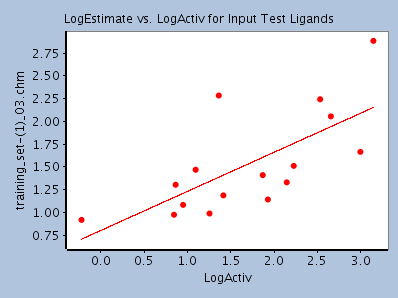

Supplement: Supplementary file 1 [file molecules-24-02870-s001.zip › Supplementary materials/3DQSARPharmacophoreGeneration/Report/images/image6082654321016201500.png]

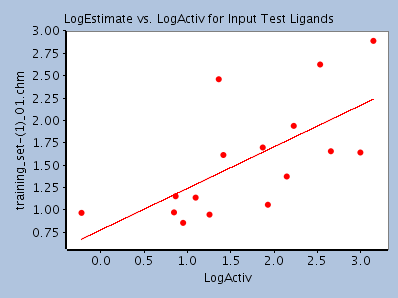

Supplement: Supplementary file 1 [file molecules-24-02870-s001.zip › Supplementary materials/3DQSARPharmacophoreGeneration/Report/images/image7126442466212802866.png]

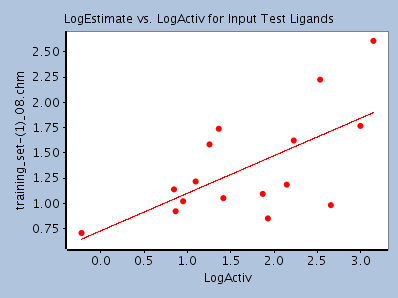

Supplement: Supplementary file 1 [file molecules-24-02870-s001.zip › Supplementary materials/3DQSARPharmacophoreGeneration/Report/images/image8881858913185323340.png]

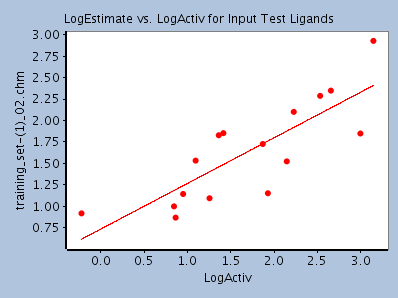

Supplement: Supplementary file 1 [file molecules-24-02870-s001.zip › Supplementary materials/3DQSARPharmacophoreGeneration/Report/images/image9033732175534185580.png]

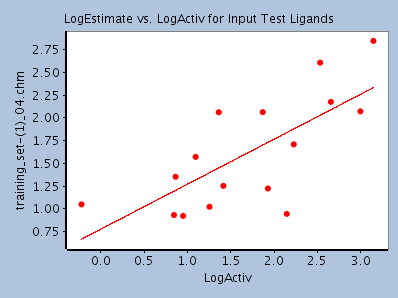

Supplement: Supplementary file 1 [file molecules-24-02870-s001.zip › Supplementary materials/3DQSARPharmacophoreGeneration/Report/images/image9101976778763198304.png]

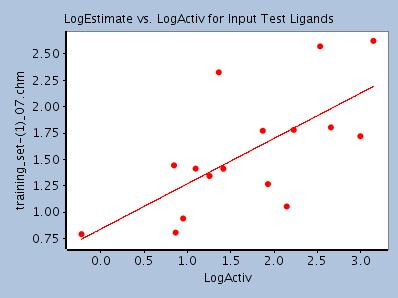

Supplement: Supplementary file 1 [file molecules-24-02870-s001.zip › Supplementary materials/3DQSARPharmacophoreGeneration/Report/images/image981321254028356053.png]
