# Supplementary material for: Discovery of Novel DPP-IV Inhibitors as Potential Candidates for the Treatment of Type 2 Diabetes Mellitus Predicted by 3D QSAR Pharmacophore Models, Molecular Docking and De Novo Evolution
Source: Molecules. 2019 Aug 7;24(16):2870. doi: 10.3390/molecules24162870 (PMC6720998; doi:10.3390/molecules24162870)
Supplement: Supplementary file 1 [file molecules-24-02870-s001.zip › Supplementary materials/Supplementary materials-final.doc]

**SUPPLEMENTARY MATERIALS**

Discovery of Novel DPP-IV Inhibitors as Potential Candidates for the Treatment of Type 2 Diabetes Mellitus predicted by 3D QSAR pharmacophore models, molecular docking and de novo evolution

Azizullo Musoev^1,2^, Sodik Numonov^1,3^, Zhuhong You^1^, Hongwei Gao^4^,*

^1^Key Laboratory of Plant Resources and Chemistry in Arid Regions, Xinjiang Technical Institute of Physics and Chemistry, Chinese Academy of Sciences, Urumqi 830011, China; [e-mail@e-azizullo.dr@gmail.com](mailto:e-mail@e-azizullo.dr@gmail.com) (A.M.); [zhuhongyou@ms.xjb.ac.cn](mailto:zhuhongyou@ms.xjb.ac.cn) (Zh.Y.)

^2^University of Chinese Academy of Sciences, Beijing, 100190, China

^3^Research Institution “Chinese-Tajik Innovation Center for Natural Products” 734063, Dushanbe, Tajikistan; [sodikjon82@gmail.com](mailto:sodikjon82@gmail.com) (S.N.)

^4^School of Life Science, Ludong University, Yantai 264025, China; [gaohongw369@ms.xjb.ac.cn](mailto:gaohongw369@ms.xjb.ac.cn) (H.G.)

* Corresponding author: Professor, Hongwei Gao, E-mail: [gaohongw369@ms.xjb.ac.cn](mailto:gaohongw369@ms.xjb.ac.cn)

Table S1: Results of the top ten pharmacophore models calculated by the HypoGen program.

| Hypo No. | Total cost | Null cost Distance | Error | RMS | Correlation | Features |
| --- | --- | --- | --- | --- | --- | --- |
| 1 | 138.152 | 352.03 | 121.61 | 2.234 | 0.925 | HBA, HBA_l, HBD, HY |
| 2 | 139.866 | 350.32 | 123.313 | 2.265 | 0.922 | HBA, HBA_l, HBD, HY |
| 3 | 164.511 | 325.67 | 147.99 | 2.665 | 0.891 | HBA_l, HBA_l, HBD, HY |
| 4 | 167.104 | 323.08 | 150.547 | 2.703 | 0.887 | HBA_l, HBA_l, HBD, HY |
| 5 | 174.539 | 315.65 | 157.633 | 2.806 | 0.878 | HBA, HBA_l, HBA_l, HY |
| 6 | 176.215 | 313.97 | 159.703 | 2.835 | 0.875 | HBA, HBA_l, HBD, HY |
| 7 | 178.056 | 312.13 | 161.417 | 2.860 | 0.873 | HBA, HBA_l, HBD, HY |
| 8 | 180.027 | 310.16 | 163.534 | 2.889 | 0.870 | HBA, HBA_l, HBD, HY |
| 9 | 192.708 | 297.48 | 176.217 | 3.060 | 0.853 | HBA, HBA_l, HBD, HY |
| 10 | 203.411 | 286.77 | 186.839 | 3.195 | 0.838 | HBA_l, HBA_l, HBD, HY |

Null cost=490.185, fixed cost=75.661, for the hypo1 weight=1.176, configuration=15.365. HBA，HBA_l, HBD，HY and HYAr are represented as hydrogen bond acceptor, hydrogen bond acceptor lipid, hydrogen bond donor, hydrophobic, and hydrophobic aromatic, respectively.

**Table S2: The interacting amino acids in the ligand-protein complex for the top 11 do**cking compounds

| Rank | Compound | Interaction amino acids |
| --- | --- | --- |
| 1 | dpp4_42 | **ARG125, GLU205, GLU206**, **TYR662,** ASP663**, TYR666,** ARG669 |
| 2 | dpp4_43 | **ARG125, GLU205, GLU206**, SER630, TYR631, VAL656, TRP659, **TYR662, TYR666,** VAL711, HIS740 |
| 3 | dpp4_44 | **ARG125**, **GLU206**, PHE357, **TYR547**, **TYR662, TYR666** |
| 4 | dpp4_45 | **ARG125, GLU206, TYR547**, **TYR662** |
| 5 | dpp4_46 | **GLU205,** SER209, **TYR662, TYR666,** ASN710**,** HIS740 |
| 6 | dpp4_47 | **ARG125**, **GLU206**, VAL207, **TYR547,** **TYR662, TYR666,** HIS740 |
| 7 | dpp4_48 | SER630**,** TYR631, VAL656, TRP659, **TYR662, TYR666,** ARG669, VAL711, HIS740 |
| 8 | dpp4_49 | **GLU206**, PHE357, **TYR547**, **TYR662, TYR666** |
| 9 | dpp4_50 | **GLU205, GLU206**, LYS554, **TYR547,** SER630, ASP663 |
| 10 | dpp4_51 | **ARG125, GLU205, GLU206**, PHE357, SER630, TYR631, **TYR662, TYR666**, HIS740 |
| 11 | Alogliptin* | **ARG125, GLU205,** PHE357, **TYR547,** TRP629, SER630, TYR631, **TYR662, TYR666** |

* represents the control compound.

**Supplemental figure captions**

**Figure S1:** Chemical structures and biological activity of the DPP-IV inhibitors in the training set.

**Figure S2:** Chemical structures and biological activity of the DPP-IV inhibitors in the test set.

**Figure S3.** Flow chart scheme of the total experimental procedures.

**Figure S1**

**Figure S2**

**Figure S3**
